# Rural Outmigration and its Double-edged Effects on Community Forestry Ecology and Governance

**DOI:** 10.1007/s00267-026-02571-5

**Published:** 2026-08-01

**Authors:** Rubina Adhikari, Truly Santika

**Affiliations:** 1 Natural Resources Institute (NRI), University of Greenwich, Chatham Maritime, ME4 4TB UK; 2 Kathmandu Forestry College (KAFCOL), M6GH+53, Chandragiri, 44600 Nepal

**Keywords:** Depopulation, Forest cover, Forest governance, Migration, Nepal, Sindhupalchowk

## Abstract

Community forestry (CF) is widely regarded as an effective decentralized governance model that delivers both social and ecological benefits. Yet, accelerating rural outmigration, driven by economic pressures and shifting generational priorities, is reshaping community demographics and challenging CF governance, a dynamic that remains empirically underexplored. Addressing this gap, this study investigates how outmigration affects forest cover, community reliance on forest resources, and the level of community participation in forestry, using both primary and secondary data from Sindhupalchowk, Nepal. Community participation in forestry is assessed across multiple dimensions, including operational activities, decision-making, and meeting attendance. The findings indicate that population decline, primarily driven by limited local livelihood opportunities, is associated with forest regeneration, suggesting that outmigration may contribute to ecological recovery. Concurrently, increasing depopulation corresponds with reduced reliance on forest resources, reflecting a broader transformation in local livelihood practices. However, this depopulation also weakens community engagement in forest management. Higher migration rates reduce participation in operational activities, decision-making, and meetings, with unemployment-driven migration having the strongest negative impact on decision-making. Overall, while reduced population pressure may support forest recovery, it simultaneously undermines local community participation, particularly in critical decision-making processes influenced by unemployment-related migration. These findings reveal a double-edged dynamic: declining populations reduce resource pressures and enable forest recovery yet concurrently weaken participatory governance and undermine the long-term socio-ecological functioning of CF systems. These findings highlight the need for adaptive strategies that align forest recovery with institutional renewal to sustain inclusive and effective CF in the context of continued demographic change.

## Introduction

Community forestry (CF) emerged in the 1970s–1980s as governments and development agencies recognized that centralized forestry had failed to curb deforestation or foster rural development. Decentralized CF offered a more inclusive model, rooted in local participation and traditional stewardship. This shift acknowledged forests’ essential role in rural livelihoods, particularly for Indigenous and local communities whose governance systems had long sustained natural resources but were often ignored by formal policy. With states unable to effectively manage vast and remote forest areas, empowering communities became both practical and necessary. The concept was crystallized at the 1978 World Forestry Congress in Indonesia, under the theme “Forests for People”. Over time, legal frameworks supporting community tenure spread across Asia, Africa, and Latin America (Gilmour 2016). Currently, approximately 14% of global forests are under community management with low- and middle-income countries accounting for 28% of this share (Hajjar et al. 2021), with increasing evidence that local stewardship can sustain ecosystems, enhance livelihoods, and advance climate goals through reduced deforestation and increased carbon sequestration (Newton et al. 2015; Pandey et al. 2016).

CF initiative has been expanding in scale and scope over the last decade, yet its long-term sustainability faces growing challenges due to shifting community livelihoods and intensified out-migration. Scholars increasingly recognize rural out-migration not merely as a demographic phenomenon but as a structural driver of land-use change shaping agricultural abandonment and the transformation of local governance and community institutions (Rudel et al. 2005; Lambin and Meyfroidt 2011). Migration can weaken commons-based institutions by reshaping local incentives and participation. In Nepal’s Mid-Hills, outmigration and livelihood diversification have substantially reduced household dependence on forest resources and diminished voluntary participation in community forestry activities (Chhetri et al. 2021). Poudyal and colleagues (2023) further suggest that this trend is fundamentally eroding the collective action that has historically underpinned successful community forest governance. A key consequence of outmigration is the reduction of agricultural labour, leading to widespread underutilisation and abandonment of farmland (Ojha et al. 2017; Maharjan et al. 2020). However, this process is uneven. Recent studies show that de-agrarianisation and re-agrarianisation occur simultaneously, with some plots abandoned while others are intensified for commercial crops or fodder trees, depending on labour availability, land quality, and market access (Poudel et al. 2024). Simultaneously, declining labour undermines landesque capital, such as terraces and irrigation systems, accelerating the transition of cultivated land into secondary forest (Marquardt et al. 2025). These dynamics link agrarian change directly to forest transition processes and reshape ecosystem functions (Marquardt et al. 2016).

Outmigration not only exerts profound impacts on rural livelihoods, but also simultaneously represents a complex socio-ecological process with both positive and negative consequences. Studies indicate that the strategic abandonment of marginal or unproductive land can reduce environmental pressures and can support soil regeneration, forest succession, and biodiversity restoration (Fischer et al. 2023; Ahmad et al. 2024). However, over the long term of diminished local governance and reduced community oversight in forest management can generate uncertainties in land tenure, creating opportunities for informal appropriation or land grabbing (Lazos-Chavero et al. 2021). Previously fragmented forests that functioned as buffers between human settlements and wildlife can become denser and more contiguous, increasing human-wildlife interactions. As forests expand into abandoned farmland, they form ecological corridors that connect habitats with settlements, reducing the capacity of local communities to protect crops and livestock (Khatri et al. 2024). This heightened interaction is associated with increased crop damage, livestock predation, and risks to human safety (Adhikari et al. 2024; Khatri et al. 2024). These interconnectedness highlights that land abandonment can foster ecological restoration, however, also fosters vulnerabilities for communities in different forms. Understanding these dynamics is essential for designing adaptive socio-ecological strategies, yet empirical evidence on these processes in the context of CF remains limited.

**Fig. 1:**
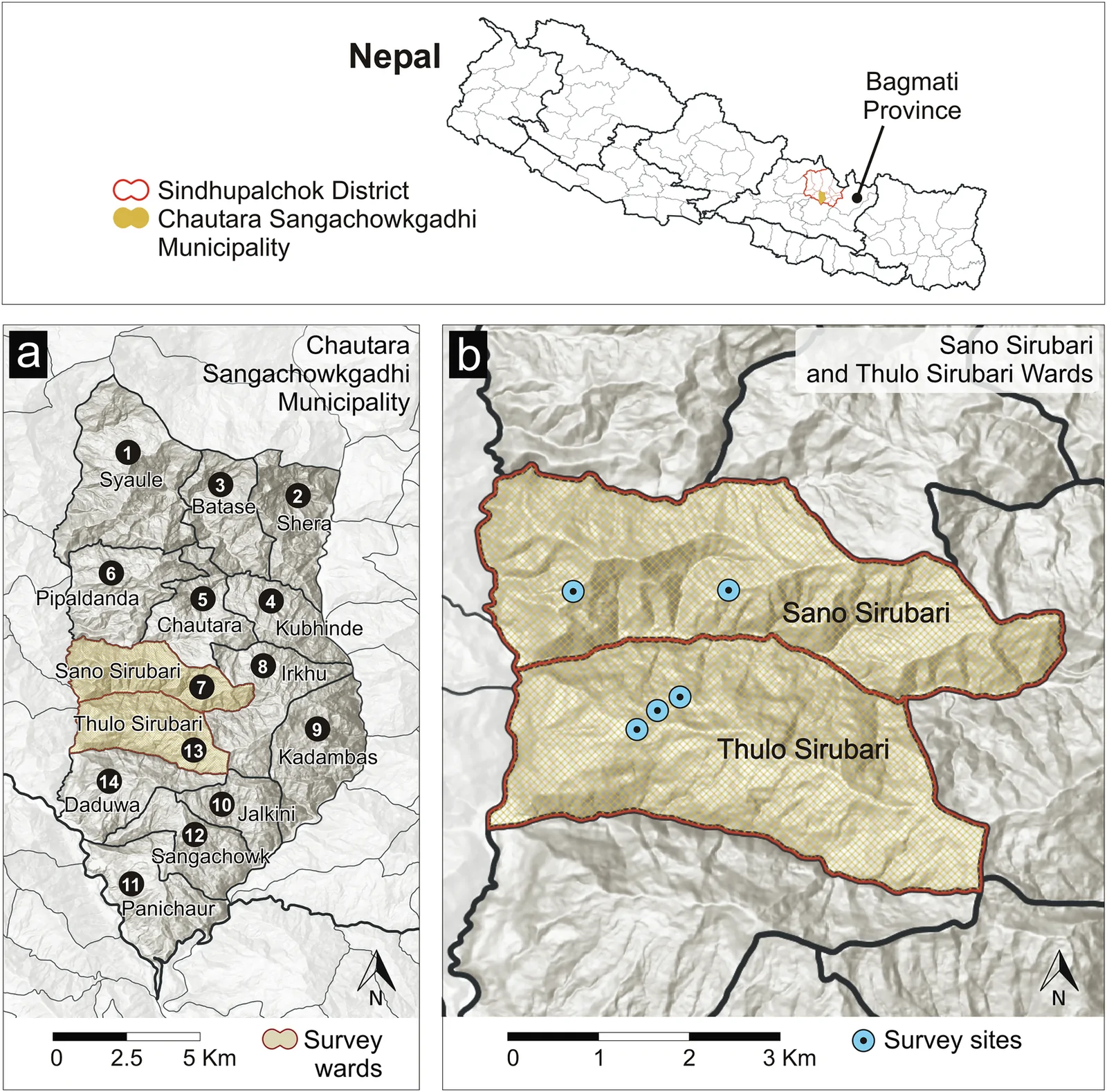
Study area showing (**a**) Chautara Sangachowkgadhi Municipality, Sindhupalchowk District, Nepal, and **b** the locations of Community Forest User Groups (CFUGs) where household surveys were conducted within Sano Sirubari and Thulo Sirubari Wards. The spatial analysis using satellite imagery and census data was carried out at the municipal scale (**a**), while household-level analysis focused on five CFUGs within area (**b**). Ward boundaries are labelled with both names and numbers, following the standard convention for designating specific geographic areas in Nepal

**Fig. 2:**
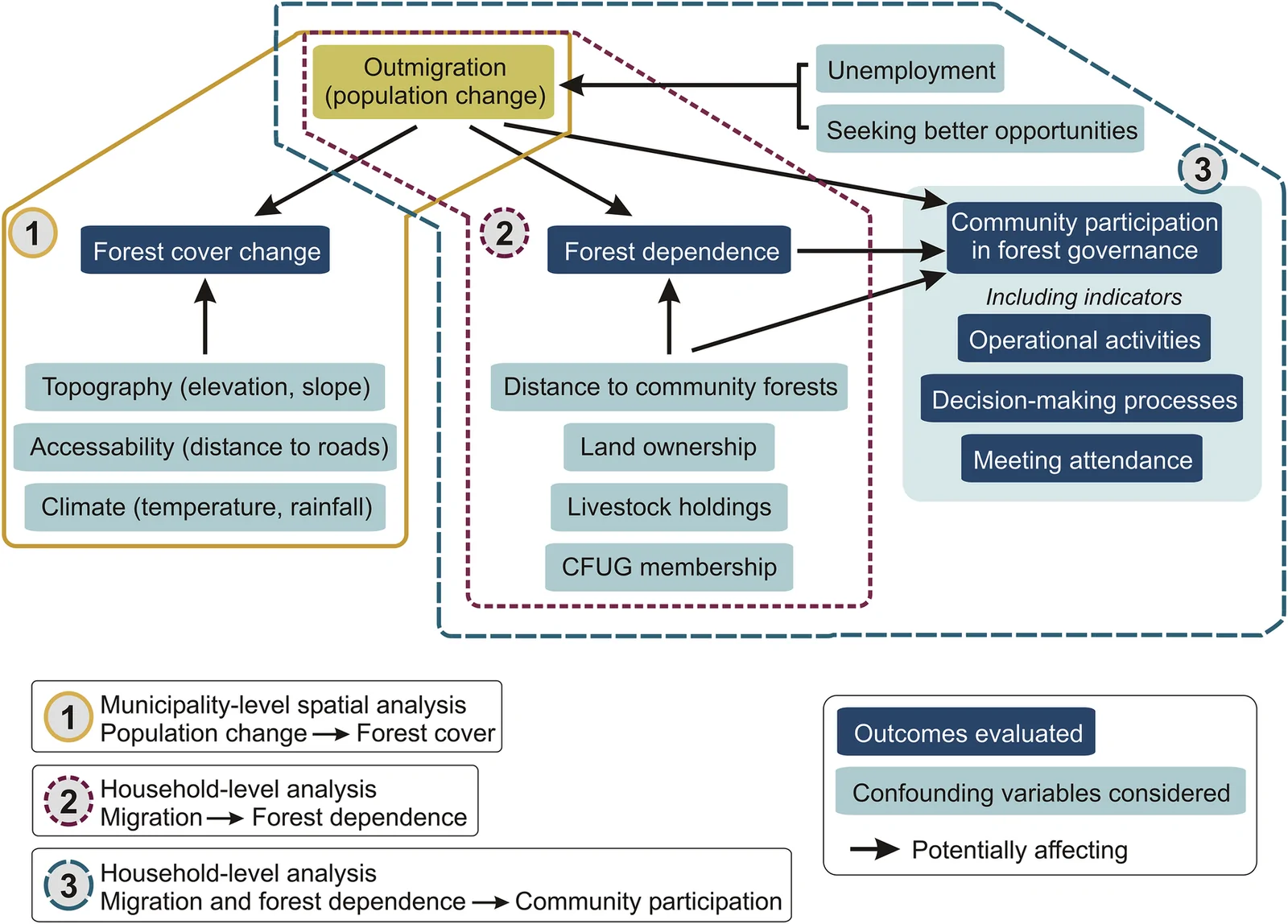
Analytical frameworks and theoretical pathways underpinning our study on outmigration, forest cover change, shifting forest dependence, and evolving community participation in forest governance. We examined three interlinked pathways: (1) how demographic shifts relate to changes in forest cover using secondary spatial datasets at the municipal level; (2) how migration influences local dependence on forest resources; and (3) how migration and changes in community dependence on forest products affect community participation in forest governance, based on primary household-level survey data

This study addresses critical gaps in existing research by examining how demographic shifts driven by outmigration influence forest cover dynamics, as well as patterns of forest dependency, livelihoods, and collective participation within CFs. While prior research has examined ecological or institutional outcomes separately (Lama et al. 2017; Bista et al. 2023), our research empirically links demographic change to both forest regrowth and participation decline within the same analytical framework. We examine Community Forest User Groups (CFUGs) in Chautara Sangachowkgadhi Municipality, Sindhupalchowk, located in Nepal’s Mid-Hills region (Fig. 1). Nepal provides an ideal context for this investigation as it currently hosts over 22,000 CFUGs, managing approximately 35% of the country’s forests, and directly engaging more than half of the population (Cadman et al. 2023; Ojha and Hall 2023). CF in Nepal has successfully reversed forest loss, with mid-hill regions shifting from annual forest decline between 1976 and 1991 to sustained growth thereafter (Tripathi et al. 2020). By integrating satellite-derived spatial data, census information, and household survey responses, this study address three interrelated questions: (1) How have demographic shifts within Chautara Sangachowkgadhi Municipality been associated with changes in forest cover? (2) In what ways has migration influenced patterns of CF utilization and altered local dependence on forest resources? (3) How have migration and changes in community dependence on forest products, in turn, shaped the extent of community participation in forest governance, particularly in terms of forest management tasks, decision-making, and leadership representation. The analytical framework linking these three interrelated questions is presented in Fig. 2. Addressing these questions enables the study to reveal the multifaceted ways migration reshapes, both directly and indirectly, the ecological, livelihood, and institutional dimensions of CF. The findings are crucial to inform adaptive strategies that can strengthen community-based forest governance amid ongoing demographic transitions.

## Community Forestry and Rural Transformations in Nepal

In Nepal, CF was formalized through the Forest Act 1993, which established CFUGs as autonomous institutions responsible for managing, conserving, and utilizing forest resources under approved operational plans (Gilmour 2016; Acharya et al. 2022; Ojha et al. 2025). This reform responded to the severe deforestation of the 1970s and has since been hailed as one of the most successful models of devolved forest governance globally. The programme has also played pivotal role in rural development channelled through social economic development. CFUG funds are often reinvested in education, irrigation, disaster recovery, and small-scale enterprises while also contributing about a quarter of household income (Bijaya et al. 2019; Nuberg et al. 2019). The Enhancing Livelihoods from Improved Forest Management in Nepal (EnLiFT) project, an ACIAR-funded research initiative operating in Sindhupalchowk among other sites, demonstrated how community-led agroforestry can improve household incomes while also supplying timber for post-disaster reconstruction (Nuberg et al. 2019). CF’s participatory frameworks have also created formal institutional mechanisms for the inclusion of women and marginalized groups, including mandatory representation provisions in CFUG committees, though the extent to which these have translated into substantive empowerment remains uneven (Ojha et al. 2025).

Despite its widely lauded achievements, Nepal’s CF continues to face persistent challenges and limitations. Unequal participation and benefit distribution is one of them where wealthier and higher caster households often dominate leadership and access to lucrative timber and other resources. On the other hand, poorer and marginalized members remain confined to service roles and receive only low value products continuing the momentum of elite capture a recurring problem in the system (Adhikari et al. 2014; Baral et al. 2019). Similarly elite capture is a recurring problem where powerful actors dominate decision-making and disproportionately benefit from forest resources. Additionally, Nepal’s federal restructuring has created overlapping responsibilities and bureaucratic inefficiencies that also hinder progress, conflicting regulations, and costly approval processes for operational plans (Acharya et al. 2022; Khatri et al. 2022). Similarly, gender inclusion, though progressive in some cases, is frequently tied to the absence of male household members rather than long-term structural change (Lama et al. 2017). Furthermore, operational plans governing CFUGs are often outdated or poorly implemented (Poudel et al. 2018). Qualitative evidence from Nepal’s mid-hills indicates declining community engagement, reflected in the lack of essential management activities and irregular institutional processes such as annual meetings and plan renewals (Poudyal et al. 2023).

In parallel, one of the most pressing challenges facing rural Nepal over the past two decades has been its dramatic demographic transformation driven primarily by the large-scale out-migration of working-age men and, increasingly, women. Economic liberalization and the lure of global labour markets have made migration a central livelihood strategy (Jaquet et al. 2016; Sunam and McCarthy 2016). By the early 2010s, nearly one-third of Nepal’s working-age male population was abroad, and remittances now contribute roughly 25–30% of GDP, reshaping rural economies and household decision-making (ILO 2017). Migration has thus become both a symptom of limited local opportunities and a structural force reconfiguring agrarian life.

## Methods

### Study Area

The Chautara Sangachowkgadhi Municipality, situated in the Sindhupalchowk District of Bagmati Province within Nepal’s Mid-Hills region (Fig. 1), is located approximately 90 km northeast of Kathmandu. Encompassing an area of about 165 km², the municipality features a diverse topography of steep hills and fertile valley floors, with an average elevation of 1600 m above sea level. According to the Department of Forest Research and Survey (DFRS 2018), approximately 8153 hectares (49.5%) of the municipality are under forest cover, predominantly managed through Nepal’s long-established CF programme. The municipality hosts 93 CF areas (Tiwari et al. 2022), reflecting robust local governance and active community engagement. The combination of substantial forest cover and strong institutional frameworks makes this region an ideal case for examining long-term landscape dynamics and participatory forest management practices.

Between the 2011 and 2021 national censuses, Chautara Sangachowkgadhi Municipality underwent a notable demographic transformation. The total population decreased from 46,497 in 2011 to 42,668 in 2021, reflecting an 8.2% decline over the decade. Despite this overall population reduction, the number of individuals absent abroad increased from 2389 to 2498, representing a 4.6% rise in international absenteeism. Concurrently, domestic absenteeism within Nepal also grew significantly, with 10,906 residents reported absent in 2021.

Our in-depth household surveys specifically targeted two wards within the municipality: Sano Sirubari and Thulo Sirubari (Fig. 1b). These wards were selected due to their high migration rates and reliance on forest resources. Sano Sirubari Ward covers 11 km², includes nine CFUGs managing 626 hectares of forest, and in 2021 had a population of 2860, with 31% reported absent. Thulo Sirubari Ward also spans 11 km², contains ten CFUGs overseeing 502 hectares, and in the same year had 3147 residents, 39% of whom were absent according to census records.

### Data

This study used two sources of data: (1) secondary spatial data derived from satellite remote sensing and official censuses, and (2) household survey data gathered during field visits. A description of these datasets is provided below and further elaborated in the Supplementary Methods.

#### Secondary Spatial Data

We utilized secondary spatial data to examine how changes in population affect forest cover (research question 1), controlling for potential confounding variables such as topography (elevation and slope), accessibility (proxied by distance to primary roads), and climate (temperature and rainfall). Our analysis specifically focused on the period between 2017 and 2024, aligning with the availability of high-resolution forest cover dataset.

##### Forest cover

Forest cover between 2017 and 2024 were extracted from Google’s Dynamic World (DW) near real-time (NRT) land use and land cover dataset. To distinguish forested areas from non-forest regions, we employed a threshold of 0.7 on the DW “Trees” class probability, consistent with thresholds validated in recent studies (Dutt et al. 2024; Zhao et al. 2025). The resulting classification closely corresponded with reported forest cover statistics for the study region. Composite imagery was generated for November, marking the onset of Nepal’s dry season, as imagery from other months was frequently affected by cloud cover and sensor limitations. Only DW composites overlapping with low-cloud ( < 5%) Sentinel-2 imagery was retained to maximize data quality. From the filtered images, an annual median composite was created, from which binary forest masks were derived (forest = 1; non-forest = 0).

##### Human population dynamics

Population data for the study years (2017 and 2024) were obtained from the WorldPop project (Tatem 2017). Preliminary analysis revealed a systematic upward bias in the raw estimates that diverged from ground-truth census records. To address this issue, the WorldPop rasters were adjusted through proportional rescaling to match the official population totals reported by the Chautara Sangachowkgadhi Municipality. Specifically, the sum of all grid-cell values within the municipality boundary was first computed, and a scaling factor was derived as the ratio between the actual municipal population and the summed WorldPop population for the same year. Each grid cell value was then multiplied by this scaling factor, ensuring that the adjusted gridded population surface preserved the spatial distribution pattern of WorldPop while matching the local population totals (Leyk et al. 2019).

##### Topography, accessibility, and climate

Topographical variation in the study area was characterized using elevation and slope. Elevation data were obtained from the Shuttle Radar Topography Mission (SRTM) at a spatial resolution of 30 metres (Farr et al. 2007). Slope was subsequently derived from the elevation data. The study area exhibits a considerable variation in elevation, ranging from 630 to 2300 m above sea level, with slope values varying between 2° and 37°. Accessibility was proxied by the Euclidean distance to primary roads. Data on road networks were obtained from open street map.

Changes in climate patterns were assessed through variations in daily temperature and annual rainfall over the study period (2017–2024). Temperature data were obtained from the ERA5 reanalysis dataset provided by the Copernicus Climate Change Service (C3S) through the Climate Data Store (Hersbach et al. 2020), while rainfall data were sourced from the CHIRPS dataset (Climate Hazards Group InfraRed Precipitation with Station data) (Funk et al. 2015).

#### Household Surveys

A total of 112 households were surveyed through a stratified random sampling strategy across five CFUGs located in Sano Sirubari and Thulo Sirubari Wards (Table S1 in the Supplementary Materials). Complementing this, five key informant interviews were conducted with CFUG leaders and other local stakeholders. Data collection was carried out over a two-week period in May 2025 by two trained enumerators working closely with the principal researcher to ensure data quality and consistency. Ethical clearance from the Research Ethics Committee of the University of Greenwich (FES-FREC-24.04.01) was obtained prior to fieldwork initiation. To facilitate comprehension and meaningful engagement, all household surveys were administered in the Nepali language, with each session lasting approximately 30 minutes. The questionnaire employed for the survey is provided in the Supplementary Data and further described below.

##### Demography and livelihoods

The survey systematically collected data on household demographics and livelihood characteristics. Demographic variables included the age, gender, caste or ethnicity of the household, along with the total number of household members. Migration patterns were also assessed, capturing both internal and international movements, their respective destinations, and the underlying motivations for migration. Additionally, households were queried about membership in CFUGs, providing insight into their engagement with local resource governance. The survey also documented the travel time required to access CF sites, providing additional context on physical accessibility and potential barriers to engagement. Land ownership was documented as the total area of land held (measured in Ropani, with 1 Ropani equivalent to 0.05 hectares) and the extent of abandoned or unused land. Livelihood indicators were further explored through the enumeration of livestock, both in aggregate livestock units and by specific categories such as goats and buffaloes.

##### Forest dependency

The survey also collected detailed information on households’ dependence on forest resources. This included primary fuel sources (e.g. LPG or firewood), the origin of firewood (whether obtained from CF areas or private land), the utilization of timber from CF areas, and the use of other non-timber forest products (NTFPs). Respondents were additionally asked to indicate whether they perceived their dependency on forest resources to have changed in the last ten years, specifying whether it had increased or decreased. Furthermore, data were collected on the current frequency of forest visits, providing insights into patterns of forest use and reliance.

##### Levels of community participation in forest governance

Efforts to conceptualize and measure participation in community forestry have consistently highlighted that it is a multi-dimensional construct, encompassing varying degrees of engagement across management, governance, and institutional processes (Chhetri et al. 2013; Adhikari et al. 2014; Oli and Treue 2015). Building on this body of literature, this study moves beyond treating participation as a single aggregate measure and instead conceptualises it as an expression of varying levels of community participation. Here, the highest level of participation refers to the capacity of households not only to take part in forest-related activities, but also to influence decisions and shape governance outcomes. It distinguishes between simply being involved and having the ability to exercise voice and influence governance outcomes, including how forest resources are managed and distributed, an important distinction in light of recent calls to reconceptualise collective action in Nepal’s community forestry under changing socio-economic conditions (Poudyal et al. 2023).

This distinction is particularly important in migration-affected contexts. Out-migration and livelihood diversification can reduce forest-people interactions, weaken social ties, and alter incentives for collective action. Empirical evidence from Nepal’s Middle Hills shows that migration is associated with declining participation in both forest management and decision-making processes (Bista et al. 2023; Laudari et al. 2024), while increasing reliance on remittances can further reduce households’ dependence on forest resources and their engagement in forest governance (Benedum et al. 2025). These changes suggest that migration may not only reduce participation overall, but also reshape who participates, how they participate, and the extent to which they can influence decisions.

To capture these distinctions, participation in this study is operationalised across three complementary domains adapted from Bista and colleagues (2023), with each domain reflecting a distinct dimension and level of community participation. These include:

(i) forest **operational activities**, which include practical tasks such as harvesting, cleaning, patrolling, and thinning that are closely tied to household dependence on forest resources. These activities sustain rural livelihoods and support timber production and other forest outputs.
(ii) **decision-making** processes, which include monitoring funds, voicing concerns, and shaping rules. This dimension is widely regarded as the most meaningful form of participation, as it reflects the ability of households to influence governance outcomes and resource allocation. It therefore serves as a key indicator of substantive empowerment and institutional inclusion.
(iii) **meeting attendance**, which is often used as a basic indicator of participation but does not necessarily imply influence over decisions. As highlighted in Agarwal’s (2001) typology, mere presence in meetings constitutes a nominal or passive form of participation, distinct from having a voice or decision-making power. Assessing meeting attendance thus allows us to distinguish between symbolic compliance and meaningful engagement, highlighting the gap between institutional presence and actual influence in forest governance.

##### Socio-ecological challenges

Survey also gathered information on socio-ecological challenges faced by the community. These challenges encompassed labour shortages, abandonment of agricultural land, and human-wildlife conflicts (HWC). Given that previous studies have identified HWC as one of the most significant challenges in CF in Nepal, we collected more detailed information on this issue. Specifically, data were obtained on the frequency of HWC incidents, the species of wildlife involved, and the direct impacts on livelihoods, including crop damage (measured in Ropani) and livestock loss.

### Statistical Analysis

Three analyses were conducted to address the study’s research questions (Fig. 2). Analysis 1 examined the association between population shifts and changes in forest cover using municipality-scale spatial datasets derived from census and satellite imagery. Analysis 2 assessed how migration influences changes in communities’ perceptions of forest dependency using household survey data. Analysis 3, drawing on the same household dataset, evaluated how changes in forest dependency affect levels of community participation. Three empirical models were applied to evaluate the relationships between key variables. An Ordinary Least Squares (OLS) model was used for Analysis 1, Generalized Additive Model (GAM) were applied in Analysis 2, and Proportional-Odds Logistic Regression (POLR) was used in Analysis 3. All variables included in the analyses are summarized in Tables S2 and S3, which reports each variable’s notation, definition and measurement, role, expected directional effect on the dependent variable, and the corresponding references.

#### Analysis 1: Population Shifts and Forest Cover Change

We investigated how shifts in population between 2017 and 2024 influenced concurrent changes in forest cover, while explicitly accounting for potential confounders such as topography, accessibility, and climate. Population dynamics serve as a proxy for varying levels of demographic pressure on forest resources. Previous research indicates that rural depopulation often promotes forest regrowth, whereas population growth tends to drive deforestation (Rudel et al. 2005; Lambin and Meyfroidt 2011). Topographic characteristics, such as elevation and slope, constrain land-use patterns, as high-altitude and steep terrains are less suitable for cultivation and are therefore more likely to remain forested, whereas accessible lowlands are subject to higher conversion pressures (Niraula et al. 2013). Proximity to roads represents a key measure of accessibility, whereby forests near roads are at greater risk of deforestation but may also benefit from lower transaction costs for community-based management interventions (Laurance et al. 2014). Climatic variability, including changes in temperature and precipitation, can also affect forest cover, with altered rainfall regimes and warming linked to forest stress and reductions in productivity (FAO 2016; IPCC 2023).

To evaluate these relationships, we analysed spatially harmonized datasets at a resolution of 1 km². Parametric OLS regression was then applied to quantify the relative contributions of demographic, while accounting for topographic, accessibility, and climatic factors, to observed forest-cover changes (Table S2). The OLS approach was selected because preliminary analyses indicated that the relationships between these variables and forest-cover change could be appropriately represented using a linear model. A detailed description of the model formulation is provided in the Supplementary Data.

#### Analysis 2: Migration and Changes in Forest Dependence

Using data from the household survey, we examined how household migration rates shape changes in perceived forest dependency, while accounting for potential confounding factors such as land and livestock ownership and proximity to forest areas. Previous studies conducted in Nepal and other regions have demonstrated that community dependence on forests varies substantially with livelihood characteristics, particularly landholding size and livestock ownership, as forests often serve as critical resources supporting these subsistence activities (Bista 2021). Similarly, the distance from households to forested areas has been shown to influence the degree of forest dependency, as greater distance can limit access to forest resources and thus modify household reliance patterns (Mendako et al. 2022).

To investigate these relationships, we employed a semi-parametric modelling framework based on GAM (Table S2). In our model, the dependent variable (*FDEP*_*i*_) represents whether a household’s forest dependency has decreased (coded as 1) or has increased or remained unchanged (coded as 0) in the past ten years The principal explanatory variable is the proportion of migrants within household *j* (*MGRT*_*j*_), calculated as the ratio of household members currently migrated to the total pre-migration household size. In addition, we incorporated a set of control variables to capture household-level characteristics that could potentially confound the relationship between migration and forest dependency. These variables include the total land area owned by the household (*LANDH*_*j*_), the number of livestock units (*LVSIZE*_*j*_), the household’s distance to the nearest CF (*CFDIST*_*j*_), and whether the household holds membership in a CFUG (*CFUG*_*j*_). Because the first three variables exhibited pronounced right-skewed distributions, we applied natural logarithmic transformations to better approximate normality, reduce the influence of extreme values, and improve the overall model fit. The data was fitted using the gam package in R (Hastie 2022), applying a logit link to appropriately model the binary response variable. A detailed description of the model specification is provided in the Supplementary Data.

#### Analysis 3: Migration, Changes in Forest Dependence, and Community Participation

Building on the analytical framework that established the relationship between household migration and shifts in forest dependency, this section extends the inquiry to examine how migration and changes in forest dependency subsequently influence levels of community participation in forest-related domains. Because migration decisions are often driven primarily by limited local employment opportunities or the pursuit of better jobs and education elsewhere (Elder et al. 2015; Jones et al. 2018), we include this factor to assess how participation varies across these motivations. To ensure robust inference, we controlled for key household- and context-level factors, including membership in CFUGs, landholding size, livestock holdings, and proximity to the CF area. Community participation was operationalized through three interrelated indicators as mentioned above.

The size of landownership and livestock holdings are widely recognized as important factors of forest participation. Landownership serves as a proxy for livelihood options and opportunity costs, with empirical evidence offering mixed insights on whether larger landholdings promote or constrain participation. Households with greater livestock endowments are often more actively engaged, reflecting enhanced labour capacity and increased demand for forest fodder (Chhetri et al. 2013; Oli and Treue 2015). Proximity to the forest area captures transaction costs and is consistently associated with participation, as households located further away are less likely to attend meetings or engage in collective activities, a pattern observed in both Nepal and Ethiopia (Chhetri et al. 2013; Tadesse et al. 2017). Beyond household and spatial factors, institutional embeddedness, measured through membership in CFUGs, remains a critical determinant of engagement. Membership confers greater access to information, decision-making authority, and associated benefits, which has been consistently linked to higher levels of participation (Oldekop et al. 2018; Bhawana and Race 2020).

To assess these relationships, we analysed the household survey data using a semi-parametric POLR model with a spline function (Table S3), implemented via the MASS package in R (Venables and Ripley 2023). This modelling approach was chosen for its flexibility in capturing potentially non-linear associations between the set of community participation indicator *m* ∈ {OP=forest operational activities, DM=decision-making, MT=meeting attendance} in household *j* (*PRTCP*_*mj*_) and both migration patterns and changes in forest dependency (*FDEP*_*j*_). Migration patterns were represented by households’ outmigration rates (*MGRT*_*j*_) and unemployment-related outmigration motives (*UNPLY*_*j*_). We also controlled for key household-level contextual variables, including landholding size (*LANDH*_*j*_), livestock units (*LVSIZE*_*j*_), distance to the CF area (*CFDIST*_*j*_), and household membership in the CFUGs (*CFUG*_*j*_). A detailed description of the model specification is provided in the Supplementary Data.

## Results

### Population Shifts and Forest Cover Change

**Fig. 3:**
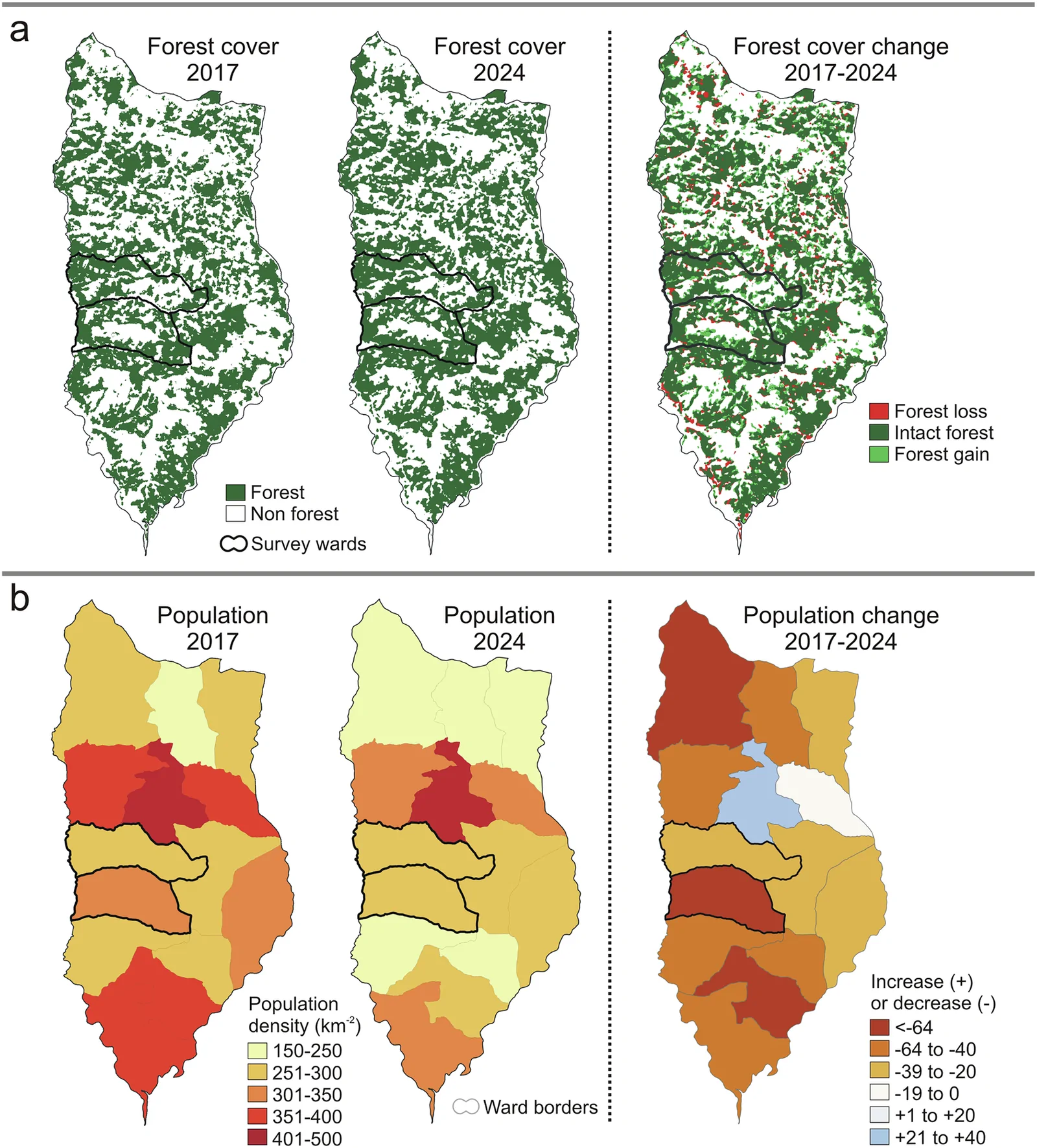
Spatial maps showing changes in (**a**) forest cover and **b** population density in Chautara Sangachowkgadhi Municipality between 2017 and 2024

Satellite data indicates that between 2017 and 2024, forest cover within Chautara Sangachowkgadhi Municipality increased by 10.2%, rising from 36.3% to 40.0% per km² on average (Fig. 3a). During the same period, the municipality’s population declined by 6.7%, with average population density decreasing from 224 to 209 people per km² (Fig. 3b).

To quantify the relative contributions of population changes to these observed forest-cover dynamics, while controlling for topography, accessibility, and climatic factors, an OLS regression was conducted. Table S4 presents the estimated coefficients of the model. The regression results indicate that population change (*ΔPOP*) is a statistically significant determinant of forest-cover dynamics (*p* = 0.017). Specifically, areas experiencing population decline exhibit greater forest regrowth, suggesting that outmigration reduces anthropogenic pressure on forest resources and facilitates natural regeneration. Baseline forest cover (*BFOR*) also shows a positive and statistically significant association with forest-cover change (*p* = 0.041), indicating that areas with higher initial forest density are more likely to experience continued regeneration. This reflects ecological persistence and the tendency of already forested areas to recover more effectively.

A detailed analysis of Sano Sirubari and Thulo Sirubari Wards further shows that forest cover in these wards increased from 1202 ha in 2017 to 1305 ha in 2024, while the total population declined from 9388 to 8791 over the same period. Building on this, household-level analysis provides deeper insights into the drivers of population change and the social mechanisms through which it reshapes forest use patterns. By focusing on migration as a key demographic process, the next section explores how shifting population dynamics are transforming community-forest relationships in the study area.

### Migration and Changes in Forest Dependence

Rural outmigration continues to significantly shape the demographic landscape of Sano Sirubari and Thulo Sirubari Wards. Surveyed households reported a total of 197 internal migrants and 55 international migrants over the past five years, resulting in an average combined migration rate of 38.3% per household. Internal migration was predominantly directed toward nearby urban centres, such as Kathmandu and Bhaktapur, driven largely by their affordability and proximity. The underlying motivations for migration shed further light on the socio-economic pressures faced by households. Among migrant households, 57.1% cited unemployment as the primary reason for leaving, highlighting the limited livelihood opportunities available locally. Meanwhile, 17.9% of households migrated to pursue higher education and 12.5% sought broader economic opportunities abroad. These patterns suggest that migration serves a dual purpose: as a coping strategy in response to local constraints and as a pathway to upward mobility. However, the predominance of economically driven migration underscores that, for most residents, leaving is more a necessity than a choice, pointing to persistent socio-economic challenges in these wards.

**Fig. 4:**
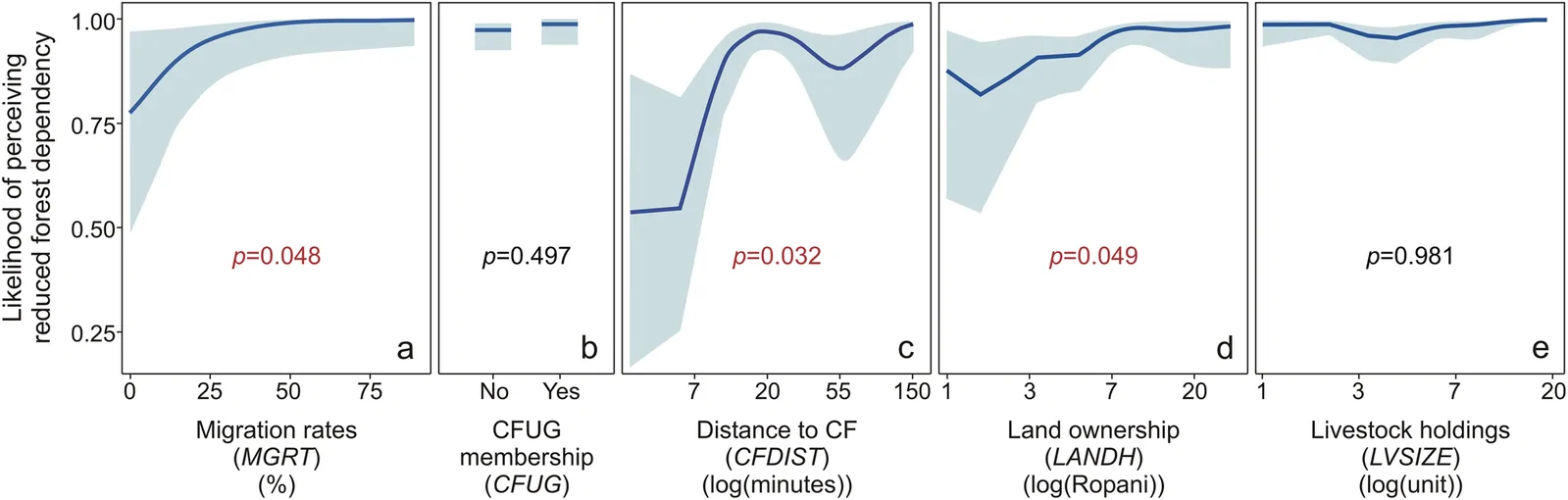
Results from the Generalized Additive Model (GAM) showing the association between household migration (*MGRT*) and the likelihood of perceiving reduced forest dependence (*FDEP*) (Eq. S2). The model accounts for key covariates, including total land area owned (*LANDH*), livestock holdings (*LVSIZE*), distance to the nearest community forest (*CFDIST*), and membership in a community forest user group (*CFUG*). Shaded areas indicate 95% confidence intervals, and red *p* values highlight variables significantly associated with lower perceived forest dependence. Detailed results are reported in Table S5

The empirical results from the GAM analysis are shown in Fig. 4 and Table S5. Migration rate (*MGRT*) has a statistically significant positive effect on the likelihood of perceived decline in forest dependency (*FDEP*) (*p* = 0.048). Specifically, a one-percent increase in migration rate is associated with a 5% higher probability that a household reported reduced dependence on forest resources. (Fig. 4a). This perception did not differ significantly between CFUG and non-CFUG members (Fig. 4b). Among the control variables, distance to the CF area (*CFDIST*) emerged as the most influential factor shaping perceptions of changing forest dependency (*p* = 0.032). Specifically, the likelihood of perceiving decreased dependency increased with greater distance from the CF (Fig. 4c), suggesting that close proximity to forests may discourage households from transitioning away from forest-based livelihoods. Land ownership (*LANDH*) also played a notable, though more moderate, role (*p* = 0.049). Households with larger landholdings exhibited a consistent tendency to perceive reduced forest dependency (Fig. 4d), possibly reflecting the availability of alternative economic opportunities that reduce reliance on forest resources. In contrast, livestock ownership showed no significant predictive power (Fig. 4e), indicating that herd size alone does not influence shifts in household forest dependency.

These findings are consistent with the descriptive evidence on energy use and resource access among community respondents. While 58% of households report firewood as their primary cooking fuel, most households adopt a mixed energy strategy, combining firewood and LPG depending on availability and household needs. Even among households that mainly used LPG, a significant share (79%) still obtained some firewood from private land, with only 21% drawing from CF. These patterns highlight the continued dominance of private land as a primary biomass source, even among LPG users. This reinforces the broader pattern of declining dependence on community forest resources and highlights a shift in livelihood practices.

### Migration, Changes in Forest Dependence, and Community Participation

**Fig. 5:**
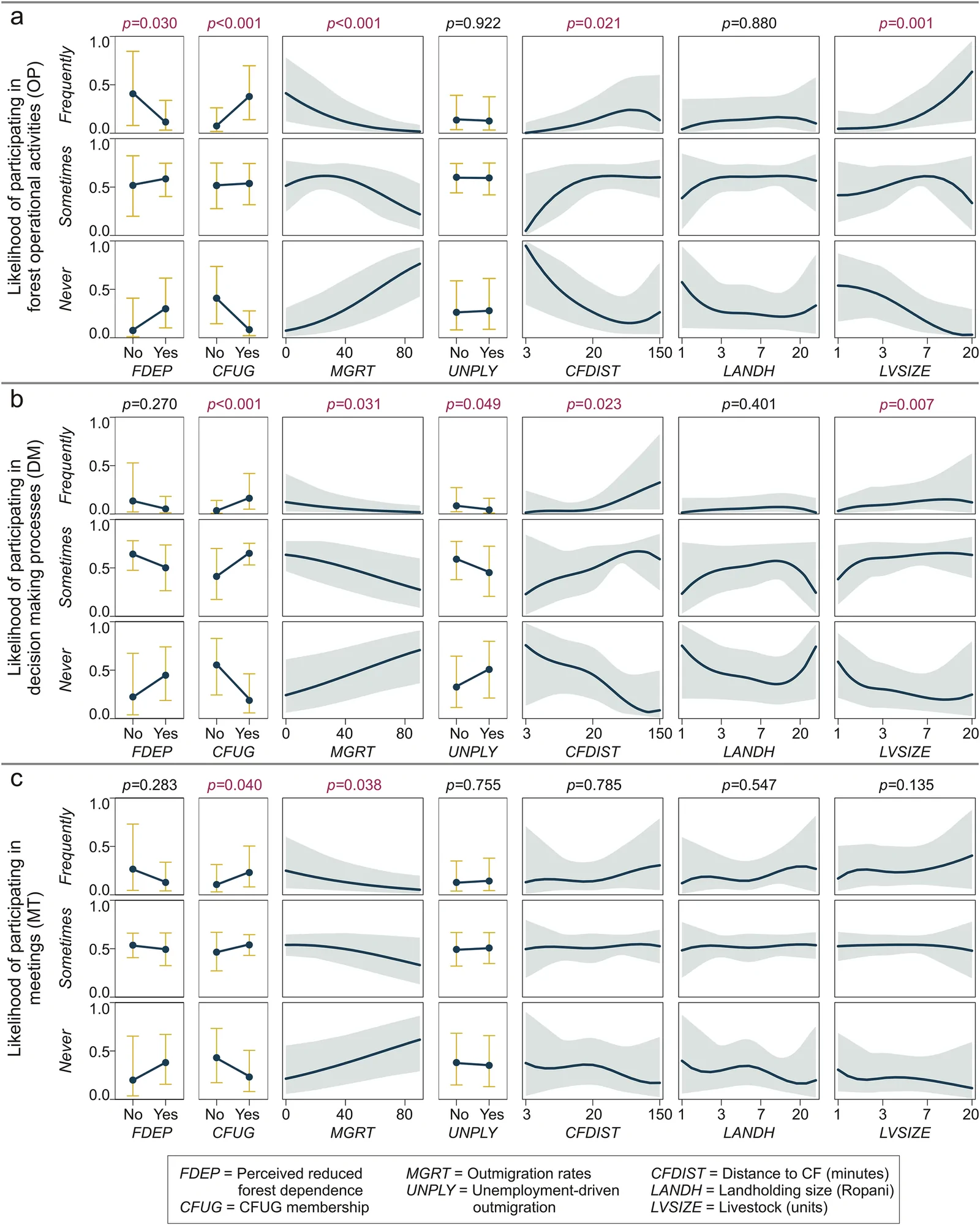
Proportional-Odds Logistic Regression (POLR) results showing how migration (*MGRT*), unemployment as a migration reason (*UNPLY*), and changes in perceived forest dependence (*FDEP*) are associated with community participation (*PRTCP*) across three dimensions: **a** forest operational activities (OP), **b** decision-making (DM), and **c** meeting attendance (MT) (Eq. S3). Each dimension was modelled separately while controlling for total land area owned (*LANDH*), livestock holdings (*LVSIZE*), proximity to the nearest community forest (*CFDIST*), and CFUG membership (*CFUG*). Shaded bands represent 95% confidence intervals, and variables with statistically significant associations are labelled with red *p*-values

Building on the conceptualisation of community participation as encompassing engagement in forest operational activities (practical tasks), decision-making (influence), and meeting attendance (institutional presence), the results reveal a clear and differentiated impact of migration on these dimensions. Figure 5 presents the results of the POLR model examining how migration, perceived forest dependence, and household characteristics influence different forms of participation in community forestry, including operational activities in Fig. 5a, decision-making in Fig. 5b, and meeting attendance in Fig. 5c.

Results show that household outmigration rates (*MGRT*) were significantly and negatively associated with all three dimensions of participation (*p* < 0.001 for operational activities, *p* = 0.031 for decision-making, and *p* = 0.038 for meeting attendance). As migration intensity increased, the probability of frequent participation declined while the probability of never participating rose. This indicates that outmigration depletes not only household labour, but also the time, local knowledge, and social networks that underpin sustained governance engagement.

Unemployment-driven migration selectively undermines decision-making. While overall migration reduced participation broadly, unemployment as a migration driver (*UNPLY*) was significantly associated only with reduced decision-making involvement (*p* = 0.049; Fig. 5b), while showing no significant effect on operational activities or meeting attendance (Fig. 5a, c). This selective pattern reflects the distinct demands of deliberative participation. Contributing to governance discussions contesting benefit distribution, proposing rule changes, influencing operational plans requires social confidence, institutional knowledge, and sufficient standing to voice preferences. Households that lost members to unemployment-driven migration are those that could not sustain livelihoods locally; their remaining members likely command fewer resources and weaker social networks, constraining their capacity to participate in deliberative settings. By contrast, routine management tasks follow pre-established rotational schedules that apply regardless of household socio-economic position, while meeting attendance is procedurally codified and often enforced through sanctions. That these households continue attending meetings while withdrawing from decision-making reveals that institutional presence can coexist with substantive disempowerment.

Declining forest dependence reduces operational engagement but not institutional involvement. Perceived reduction in forest dependence (*FDEP*) was significant for operational activities (*p* = 0.030; Fig. 5a), but not for decision-making or meeting attendance (Fig. 5b, c). As the material value of forest resources diminishes relative to alternative income sources such as remittances, households reduce investment in labour-intensive management tasks. That decision-making and meeting attendance remain unaffected suggests these forms of engagement are sustained by motivations beyond direct resource use including social belonging, tenure security, and community obligation.

Covariates confirm the role of institutional membership, spatial access, and livelihood stakes. CFUG membership (*CFUG*) was significant across all three dimensions (*p* < 0.001 for forest management and decision-making, and *p* = 0.040 for meeting attendance; Fig. 5a–c), confirming formal membership as a prerequisite for engagement. Distance to community forest (*CFDIST*) was significant for management (*p* = 0.021; Fig. 5a) and decision-making (*p* = 0.023; Fig. 5b), reflecting spatial transaction costs that compound constraints on peripheral households. Livestock holdings (*LVSIZE*) were significant for management (*p* = 0.001; Fig. 5a) and decision-making (*p* = 0.007; Fig. 5b), consistent with stronger participatory incentives among fodder-dependent households. Landholding size (*LANDH*) showed no significant association with any dimension.

These findings collectively imply that outmigration does not erode community participation uniformly. High overall migration reduces participation across all governance dimensions, but unemployment-driven migration selectively undermines decision-making, the domain where governance priorities are set and resource distribution is negotiated. Routine forest management activities and meeting attendance, governed by institutional obligations and lower participation thresholds, remain comparatively unaffected. This divergence highlights the importance of differentiating meaningful participation, and that attendance-based assessments of participatory may overestimate the substantive influence of migration-affected households. Households may fulfil procedural requirements such as attending meetings and participating in scheduled management tasks while lacking the social and economic capital to shape the governance decisions that determine how forest benefits are distributed.

## Discussion

Outmigration has altered patterns of forest use and participation, producing a paradox of ecological recovery alongside social disengagement. This dual process underscores the complex feedback between demographic change and environmental governance, challenging simplistic narratives of forest recovery as an unqualified success. The subsequent discussion unpacks these dynamics as they manifest within the Chautara Sangachowkgadhi Municipality, situating them within broader literature. This paradox is increasingly recognized in agrarian and forest-transition scholarship, where forest recovery is often accompanied by weakening collective action due to shifting livelihood systems and declining dependence on subsistence forest use (Poudyal et al. 2023).

### Demographic Change and Forest Cover Dynamics

Our analysis reveals a significant association between population decline and forest cover gain, confirming that the ecological landscape of the study area is undergoing active regeneration. This trend aligns with the broader trajectory of forest transition observed in Nepal’s Mid-Hills, where depopulation and reduced dependence on subsistence agriculture have facilitated secondary forest expansion (Oldekop et al. 2018; Bhawana and Race 2020). Importantly, this local pattern is part of a larger global phenomenon of post-agricultural reforestation. For instance, rural out-migration in mid-20th century Puerto Rico led to widespread farmland abandonment and a rise in forest cover from 9% in 1950 to 37% by 1990 (Aide and Grau 2004). Similar upland reforestation has been documented in depopulated regions of Spain and France (Mather and Needle 2000). However, the relationship between migration and forest regrowth is not universally positive. Contextual factors critically shape its trajectory, particularly over the long term. In Mexico, for example, land abandonment facilitated forest regrowth in some areas, while remittance inflows financed extensive cattle ranching in others, accelerating deforestation (Ervin et al. 2020). Likewise, in the Amazon, rural depopulation coincided not with forest recovery but with the expansion of commercial agribusiness into depopulated landscapes (Rudel et al. 2005). In our study landscape, although depopulation has had a positive impact on forest cover, it simultaneously poses critical challenges to the socio-ecological and institutional capacity of CF programmes, highlighting the need for forward-looking governance strategies.

Migration has emerged as a central driver of this transition, reshaping household economies through remittance income, diversification into non-farm activities, and reduced dependence on subsistence agriculture. These findings are consistent with earlier studies showing that economic modernisation and the adoption of alternative energy sources tend to weaken local engagement with forest resources (Adhikari et al. 2014; Oli and Treue 2015; Poudyal et al. 2023). National energy transitions support the shift as the reliance on firewood declined from 64 to 51%, while the use of LPG increased from 21 to 44% between 2011 and 2021 (National Statistics Office 2023). This change reflects not only a technological transition but also a deeper behavioural and cultural shift, from subsistence-oriented practices toward consumption patterns driven by remittance income and stronger market integration. Comparable trends have been observed in other Mid-Hill regions of Nepal, where migration and remittance economies have restructured local labour markets and reduced participation in forest management activities (Bista et al. 2023). Simultaneously, a growing dependence on alternative energy sources further diminishes incentives for active participation in forest stewardship (Marquardt et al. 2016).

Cook and colleagues (2025) provide a theoretical framework for understanding these shifts through a cost-benefit lens arguing that as households raise their incomes through out-migration and livelihood diversification, the opportunity costs associated with CF participation increase. This reasoning is directly corroborated by our survey findings, where respondents consistently reported declining interest in forest product collection alongside rising engagement in off-farm income activities. Furthermore, the declining reliance on community forest products is tightly linked to changing agricultural practices. As Chhetri and colleagues (2021) observe, farmers in their study sites halved their ownership of large livestock between 1992 and 2017 and diversified into smaller monogastric animals (poultry, goats), dramatically reducing demand for fodder and leaf litter from community forests. Poudyal and colleagues (2023) found an analogous pattern, with approximately 85% of respondents in mountain study villages reporting declining frequency of forest product collection and use over the preceding decade. Collectively, these dynamics suggest that while migration-induced economic mobility enhances household welfare, it simultaneously erodes the social reciprocity and collective engagement that form the foundation of participatory forest governance.

### Institutional Erosion and the Limits of Participatory Governance

Although CFUG committee members remain active in leadership and decision-making roles, this selective participation risks creating institutional imbalance. Membership has become largely symbolic, as the declining dependence on forests no longer provides a meaningful basis for engagement, undermining both democratic legitimacy and social trust (Robson and Berkes 2011). This pattern mirrors broader evidence that nominal participation, such as attending meetings or holding membership, does not necessarily equate to meaningful influence in governance (Oli and Treue 2015). This institutional decay was also reflected in the status of operational plans in our study area, which were often outdated or exhibited declining implementation over time, a trend similarly identified by Poudyal and colleagues (2023).

The concentration of authority in the hands of a few individuals not only overburdens these leaders but also erodes the participatory foundations upon which CF was originally built (Pariyar et al. 2025). Yet, with most active members being older, the institutional base is likely to weaken further without generational renewal and stronger youth engagement. The perpetual decline in the participation in forest management activities, disrupts the intergenerational transmission of forest knowledge and customary practices. This gradual disconnection threatens the cultural foundations of CF itself (Robson and Klooster 2019). As Brown (2021) cautions, unless governance institutions actively foster inclusive spaces for youth and women, demographic shifts will deepen existing exclusions, transforming CFUGs into aging bureaucratic entities rather than adaptive, community-based institutions capable of renewal.

### Ecological Implications: Forest Quality and Human-wildlife Conflict

In parallel, passive reforestation raises questions of forest quality as much as forest quantity. Although canopy cover has expanded in recent decades, research cautions that secondary forests often comprise fast-growing, low-diversity species that deliver limited biodiversity benefits (Robson and Berkes 2011). Furthermore, inadequate silvicultural management, minimal fire-prevention efforts within CFUGs, and uncontrolled biomass accumulation have heightened the risk of severe forest fires (Tiwari et al. 2022). As farming becomes less viable due to labour shortages and declining returns, marginal lands are progressively abandoned, facilitating forest regeneration. At the same time, these abandoned lands alter landscape structure by expanding forest edges and creating new transitional zones between forests and settlements. Importantly, such changes are not ecologically neutral. Emerging evidence from Nepal’s Himalayan farming landscapes shows that abandoned farmlands often evolve into secondary forest patches and ecological corridors, which provide shelter and movement pathways for wildlife (Khatri et al. 2024).

An unintended outcome of these ecological shifts has been the escalation of human-wildlife conflict (HWC). Survey responses and incident reports frequently cited crop raids by monkeys and wild boars, reflecting a growing tension between regenerating forests and nearby agricultural lands. This intensification appears closely tied to forest regeneration and declining human activity, both of which have narrowed the buffer zone between croplands and wildlife habitats (Bista and Song 2022). National assessments similarly attribute the rising incidence of HWC to habitat reconnection, farmland abandonment, and ineffective compensation mechanisms that exclude common species while demanding burdensome documentation (Bhushal et al. 2024). Yet, farmland abandonment itself is not only a passive response to ecological change as declining household labour limits the ability to cultivate distant plots or guard crops, making agriculture increasingly unviable (Marquardt et al. 2025).

The current compensation policy offers a flat rate of NPR 10,000 ( ~ USD 70) per HWC incident, regardless of damage extent, yet requires the submission of around ten supporting documents, a process inaccessible to many rural households. Although legislation permits the culling of wild boars, confronting herds of 8-10 animals at night demands firearms and training that most elderly villagers understandably lack. Consequently, many communities remain trapped between mounting crop and livestock losses and the absence of practical institutional support, revealing the complex trade-offs embedded in passive reforestation strategies. On one hand, out-migration can relieve pressure on agricultural lands, allowing for natural reforestation and aligning with global narratives of rural depopulation and ecological recovery (Meyfroidt et al. 2018). On the other hand, the departure of rural labour disrupts institutional continuity, erodes social cohesion, and increases the risk of unmanaged landscapes, potentially producing ecological and social outcomes that are uncertain and difficult to predict.

## Conclusion

This study examined how rural outmigration affects the ecological, livelihood, and institutional dimensions of CF using spatial and household survey data from Sindhupalchowk, Nepal. Three empirical contributions emerge from this analysis. **First**, population decline is significantly associated with forest cover gain, confirming that migration-mediated depopulation enables natural regeneration. However, this ecological recovery is passive and unmanaged. The forests that are expanding are denser but potentially less diverse, less actively maintained, and more prone to fire and human-wildlife conflict which raises critical concerns about whether quantity of forest cover can substitute for quality of forest governance. **Second**, migration significantly increases the likelihood that households perceive declining dependence on forest resources. This shift is reinforced by energy transitions from firewood to LPG, reduced livestock holdings, and the growing primacy of remittance-based and off-farm income. As the material incentives for engaging with community forests weaken, the social contract underpinning collective forest management frays. Notably, this decline in perceived dependency does not differ between CFUG members and non-members, suggesting that the erosion of forest-livelihood linkages is systemic rather than institutional. **Third**, and most critically, the study demonstrates that outmigration does not erode community participation uniformly. While higher migration rates reduce community participation across all dimensions, unemployment-driven migration selectively undermines decision-making. That households continue attending meetings while withdrawing from substantive deliberation exposes a troubling gap between institutional presence and meaningful influence. Attendance-based metrics, commonly used to assess participatory status, may therefore overestimate the democratic vitality of migration-affected CFUGs.

These dynamics carry direct implications for Nepal’s forest policy architecture. The Forest Act of 1993, the revised Act of 2019, and the Forestry Sector Strategy (2016-2025) were designed for a context of high rural population density and strong local dependence on forest resources, conditions that no longer hold in many Mid-Hill communities. The current policy framework does not account for the demographic realities of mass outmigration, declining forest dependency, or the differentiated erosion of community participation and influence documented here. Embedding demographic considerations into forest governance recognizing absentee populations, adapting participation requirements, and aligning institutional expectations with actual community capacity is no longer optional but essential.

Simultaneously, this study’s scope necessarily leaves several questions open. How might CFUG institutional structures be reorganized to remain functional amid depopulation? What mechanisms could meaningfully re-engage youth and returned migrants in forest governance, and under what conditions would remittance flows translate into productive investment in forest-based enterprises rather than further disengagement? These are imperative questions that demand dedicated investigation.

In summary, our findings reveal a central paradox: outmigration reduces anthropogenic pressure and facilitates forest regrowth yet simultaneously erodes the participatory foundations upon which CF was built. These findings challenge the prevailing narrative that forest recovery signals conservation success. In the study area, ecological gains and institutional losses are two sides of the same demographic process. The forests are growing, but the communities mandated to govern them are shrinking, aging, and disengaging. Left unaddressed, this trajectory risks producing the ironic outcome of expanding forests overseen by hollowed-out institutions that are ecologically present but governmentally absent. Accordingly, CF can no longer be assessed through ecological indicators alone. The challenge for Nepal is to develop governance frameworks that can adapt to ongoing demographic changes. CF was originally designed under subsistence-based conditions, but it now operates in a context shaped by migration and diversified livelihoods. Its future relevance will depend on whether it can evolve to sustain both forest conservation and effective community participation under these changing conditions.

## Supplementary information


SupplementaryMaterial


## Data Availability

All data used in this study are available upon request from the corresponding author.
