# Supplementary material for: Rural Outmigration and its Double-edged Effects on Community Forestry Ecology and Governance
Source: Environ Manage. 2026 Aug 1;76(8):260. doi: 10.1007/s00267-026-02571-5 (PMC13428711; doi:10.1007/s00267-026-02571-5)
Supplement: Supplementary file 1 — SupplementaryMaterial [file 267_2026_2571_MOESM1_ESM.docx]

**Supplementary Materials**

Rural outmigration and its double-edged effects on community forestry ecology and governance

Rubina Adhikari ^1,2^ & Truly Santika ^1,^*

**This file includes:**

Supplementary Methods (pages 1-7)

Supplementary Data: Survey Questionnaire (pages 8-11)

Supplementary Tables: Table S1-S5 (pages 12-16)

Supplementary Figures: Figure S1 (page 17)

Supplementary References (pages 18-20)

______________________________________

*Affiliations:*

1. Natural Resources Institute (NRI), University of Greenwich, Chatham Maritime ME4 4TB, UK
2. Kathmandu Forestry College (KAFCOL), M6GH+53, Chandragiri 44600, Nepal

* Corresponding author: Truly Santika ([T.Santika@greenwich.ac.uk](mailto:T.Santika@greenwich.ac.uk))

Supplementary Methods

This section provides a comprehensive description of the methodology used in this study. It covers: (a) the data sources, including (i) secondary spatial data and (ii) household survey data collected through fieldwork; and (b) the statistical analyses conducted, including (i) the analysis of population shifts and forest cover change, (ii) the analysis of migration and changes in forest dependence, and (iii) the analysis of migration, changes in forest dependence, and community participation.

1. **Data**

This study used two sources of data: (1) secondary spatial data derived from satellite remote sensing and official censuses, and (2) household survey data gathered during field visits. A detailed description of these datasets is provided below.

A.1. Secondary spatial data

We utilized secondary spatial data to examine how changes in population affect forest cover (research question 1), controlling for potential confounding variables such as topography (elevation and slope), accessibility (proxied by distance to primary roads), and climate (temperature and rainfall). Our analysis specifically focused on the period between 2017 and 2024, aligning with the availability of high-resolution forest cover dataset.

*A.1.1. Forest cover*

Forest cover between 2017 and 2024 were extracted from Google’s Dynamic World (DW) near real-time (NRT) land use and land cover dataset. DW dataset provides global coverage at 10-meter resolution and frequent temporal updates aligned with Sentinel-2’s 2-5-day revisit cycle (Brown et al. 2022). The dataset generates continuous probabilistic land classifications, enabling precise detection of changes over time and supporting confidence-based masking. Compared to other land cover datasets, DW has demonstrated superior performance in identifying fine-scale alterations, particularly in transitional landscapes where forests are subject to rapid fragmentation, regrowth, or other dynamic processes (Venter et al. 2022).

To distinguish forested areas from non-forest regions, we employed a threshold of 0.7 on the DW “Trees” class probability, consistent with thresholds validated in recent studies (Dutt et al. 2024; Zhao et al. 2025). The resulting classification closely corresponded with reported forest cover statistics for the study region. Composite imagery was generated for November, marking the onset of Nepal’s dry season, as imagery from other months was frequently affected by cloud cover and sensor limitations. Only DW composites overlapping with low-cloud (<5%) Sentinel-2 imagery was retained to maximize data quality. From the filtered images, an annual median composite was created, from which binary forest masks were derived (forest=1; non-forest=0).

*A.1.2. Human population dynamics*

Population data for the study years (2017 and 2024) were obtained from the WorldPop project (Tatem 2017). Preliminary analysis revealed a systematic upward bias in the raw estimates that diverged from ground-truth census records. Previous research by Láng-Ritter and colleagues (2025) demonstrates that global population datasets such as WorldPop often misrepresent rural areas, with both under- and overestimations arising from constraints in input data and disaggregation methods. To address this issue, the WorldPop rasters were adjusted through proportional rescaling to match the official population totals reported by the Chautara Sangachowkgadhi Municipality. Specifically, the sum of all grid-cell values within the municipality boundary was first computed, and a scaling factor was derived as the ratio between the actual municipal population and the summed WorldPop population for the same year. Each grid cell value was then multiplied by this scaling factor, ensuring that the adjusted gridded population surface preserved the spatial distribution pattern of WorldPop while matching the local population totals. Such proportional rescaling of a gridded population surface to match authoritative local totals aligns with recommended methodological practices in the use of global population grids (Leyk et al. 2019).

*A.1.2. Topography, accessibility, and climate*

Topographical variation in the study area was characterized using elevation and slope. Elevation data were obtained from the Shuttle Radar Topography Mission (SRTM) at a spatial resolution of 30 meters (Farr et al. 2007). Slope was subsequently derived from the elevation data. The study area exhibits a considerable variation in elevation, ranging from 630 m to 2300 m above sea level, with slope values varying between 2° and 37°. Accessibility was proxied by the Euclidean distance to primary roads. Data on road networks were obtained from open street map.

Changes in climate patterns were assessed through variations in daily temperature and annual rainfall over the study period (2017-2024). Temperature data were obtained from the ERA5 reanalysis dataset provided by the Copernicus Climate Change Service (C3S) through the Climate Data Store (Hersbach et al. 2020), while rainfall data were sourced from the CHIRPS dataset (Climate Hazards Group InfraRed Precipitation with Station data) (Funk et al. 2015). Analysis indicates that the mean daily temperature within the study area was approximately 17°C in 2017, rising by 0.7°C by 2024. Likewise, annual rainfall rose from an estimated 1,385 mm in 2017 to a value roughly 12.2% higher in 2024. These results suggest a clear warming trend accompanied by increased precipitation in the study area over the observed period.

A.2. Household surveys

A total of 112 households were surveyed through a stratified random sampling strategy across five CFUGs located in Sano Sirubari and Thulo Sirubari Wards (Table S1). Complementing this, five key informant interviews were conducted with CFUG leaders and other local stakeholders. Data collection was carried out over a two-week period in May 2025 by two trained enumerators working closely with the principal researcher to ensure data quality and consistency. The research protocol received ethical clearance from the Ethics Committee of the researchers’ institution prior to fieldwork initiation.

To facilitate comprehension and meaningful engagement, all household surveys were administered in the Nepali language, with each session lasting approximately 30 minutes. The survey instrument captured information on key domains, including household demographics, livelihood strategies, levels of community participation in forest governance, and patterns of forest use and dependence. The questionnaire employed for the survey is provided in the Supplementary Data and further described below.

*A.2.1. Demography and livelihoods*

The survey systematically collected data on household demographics and livelihood characteristics. Demographic variables included the age, gender, caste or ethnicity of the household, along with the total number of household members. Migration patterns were also assessed, capturing both internal and international movements, their respective destinations, and the underlying motivations for migration. Additionally, households were queried about membership in CFUGs, providing insight into their engagement with local resource governance. The survey also documented the travel time required to access CF sites, providing additional context on physical accessibility and potential barriers to engagement.

Land ownership was documented in detail, including the total area of land held (measured in Ropani, with 1 Ropani equivalent to 0.05 hectares) and the extent of abandoned or unused land. Livelihood indicators were further explored through the enumeration of livestock, both in aggregate livestock units and by specific categories such as goats and buffaloes.

*A.2.2. Forest dependency*

The survey also collected detailed information on households’ dependence on forest resources. This included primary fuel sources (e.g. LPG or firewood), the origin of firewood (whether obtained from CF areas or private land), the utilization of timber from CF areas, and the use of other non-timber forest products (NTFPs). Respondents were additionally asked to indicate whether they perceived their dependency on forest resources to have changed in the last ten years, specifying whether it had increased or decreased. Furthermore, data were collected on the current frequency of forest visits, providing insights into patterns of forest use and reliance.

*A.2.3. Levels of community participation in forest governance*

Efforts to conceptualize and measure participation in community forestry have consistently highlighted that it is a **multi-dimensional construct**, encompassing varying degrees of engagement across management, governance, and institutional processes. Previous studies have applied ordered probit and logit models to capture household-level variation in participation across forest management and governance activities (Chhetri et al. 2013; Oli & Treue 2015). Others have developed composite indices using factor analysis or Likert-scale approaches, incorporating dimensions such as meeting attendance, decision-making involvement, implementation of operational plans, and benefit-sharing (Adhikari et al. 2014; Dhungana et al. 2024). In Ethiopia, participation has been operationalized through planning, implementation, and monitoring indices, further underscoring the importance of evaluating engagement across multiple stages of participatory forest management (Tadesse et al. 2017). More recent work in Nepal distinguishes between active and passive forms of participation, showing that limited economic returns, particularly under protection-oriented forest management can reduce incentives for sustained engagement, especially in migration-affected contexts (Pariyar et al. 2025).

**Building on this literature, this study moves beyond treating participation as a single aggregate measure and instead conceptualises it as an expression of varying levels of** community participation**.** Here, the highest level of participation refers to the **capacity of households not only to take part in forest-related activities, but also to influence decisions and shape governance outcomes**.

Building on this literature, this study moves beyond treating participation as a single, aggregate construct by conceptualising it as a continuum of community participation. At the highest level, participation extends beyond involvement in forest-related activities to encompass households' capacity to influence decision-making and shape governance outcomes. In other words, it distinguishes between simply being involved and having the ability to exercise voice and influence governance outcomes, including how forest resources are managed and distributed, an important distinction in light of recent calls to reconceptualise collective action in Nepal’s community forestry under changing socio-economic conditions (Poudyal et al. 2023).

This distinction is particularly important in migration-affected contexts. Out-migration and livelihood diversification can reduce forest–people interactions, weaken social ties, and alter incentives for collective action. Empirical evidence from Nepal’s Middle Hills shows that migration is associated with declining participation in both forest management and decision-making processes (Bista et al. 2023; Laudari et al. 2024), while increasing reliance on remittances can further reduce households’ dependence on forest resources and their engagement in forest governance (Benedum et al. 2025). These changes suggest that migration may not only reduce participation overall, but also reshape **who participates, how they participate, and the extent to which they can influence decisions**.

To capture these distinctions, participation in this study is operationalised across three complementary domains adapted from Bista and colleagues (2023), with each domain reflecting a distinct dimension and level of community participation. These include:

1. **forest operational activities**, which include practical tasks such as harvesting, cleaning, patrolling, and thinning that are closely tied to household dependence on forest resources. These activities sustain rural livelihoods and support timber production and other forest outputs.
2. **decision-making processes**, which include monitoring funds, voicing concerns, and shaping rules. This dimension is widely regarded as the most meaningful form of participation, as it reflects the ability of households to influence governance outcomes and resource allocation. It therefore serves as a key indicator of substantive empowerment and institutional inclusion.
3. **meeting attendance**, which is often used as a basic indicator of participation but does not necessarily imply influence over decisions. As highlighted in Agarwal’s (2001) typology, mere presence in meetings constitutes a nominal or passive form of participation, distinct from having a voice or decision-making power. Assessing meeting attendance thus allows us to distinguish between symbolic compliance and meaningful engagement, highlighting the gap between institutional presence and actual influence in forest governance.

*A.2.4. Socio-ecological challenges*

Survey also gathered information on socio-ecological challenges faced by the community. These challenges encompassed labour shortages, abandonment of agricultural land, and human-wildlife conflicts (HWC). Given that previous studies have identified HWC as one of the most significant challenges in CF in Nepal, we collected more detailed information on this issue. Specifically, data were obtained on the frequency of HWC incidents, the species of wildlife involved, and the direct impacts on livelihoods, including crop damage (measured in Ropani) and livestock loss.

1. **Statistical analysis**

Three analyses were conducted to address the study’s research questions (Figure 2). Analysis 1 examined the association between population shifts and changes in forest cover using municipality-scale spatial datasets derived from census and satellite imagery. Analysis 2 assessed how migration influences changes in communities’ perceptions of forest dependency using household survey data. Analysis 3, drawing on the same household dataset, evaluated how changes in forest dependency affect levels of community participation. Three empirical models were applied to evaluate the relationships between key variables. An Ordinary Least Squares (OLS) model was used for Analysis 1, Generalized Additive Model (GAM) were applied in Analysis 2, and Proportional-Odds Logistic Regression (POLR) was used in Analysis 3. All variables included in the analyses are summarized in Tables S2 and S3, which reports each variable’s notation, definition and measurement, role, expected directional effect on the dependent variable, and the corresponding references.

OLS is a parametric model, whereas GAM and POLR are semi-parametric models. Parametric models impose a fixed functional form, such as a linear specification, while semi-parametric models combine parametric components with flexible nonparametric smoothing techniques, such as splines, to capture unknown functional relationships (Hastie 2017). These differences affect how results are reported and interpreted. In parametric models, coefficients are directly interpreted as marginal effects, while in semi-parametric models, linear terms are interpreted in a similar way, whereas smooth terms are typically presented visually rather than as numerical coefficients (Hastie 2017). Accordingly, the results of Analysis 1 are primarily presented in tabular form (Table S4) and supplemented by spatial visualisations (Figure 3), whereas those of Analysis 2 and Analysis 3 are mainly presented using plots of marginal effects (Figures 4 and 5, respectively). Detailed descriptions of each analysis are provided in the following sections.

B.1. Analysis 1: Population shifts and forest cover change

We investigated how shifts in population between 2017 and 2024 influenced concurrent changes in forest cover, while explicitly accounting for potential confounders such as topography, accessibility, and climate. Population dynamics serve as a proxy for varying levels of demographic pressure on forest resources. Previous research indicates that rural depopulation often promotes forest regrowth, whereas population growth tends to drive deforestation (Rudel et al. 2005; Lambin & Meyfroidt 2011). Topographic characteristics, such as elevation and slope, constrain land-use patterns, as high-altitude and steep terrains are less suitable for cultivation and are therefore more likely to remain forested, whereas accessible lowlands are subject to higher conversion pressures (Niraula et al. 2013). Proximity to roads represents a key measure of accessibility, whereby forests near roads are at greater risk of deforestation but may also benefit from lower transaction costs for community-based management interventions (Laurance et al. 2014). Climatic variability, including changes in temperature and precipitation, can also affect forest cover, with altered rainfall regimes and warming linked to forest stress and reductions in productivity (FAO 2016; IPCC 2023).

To evaluate these relationships, we analysed spatially harmonized datasets at a resolution of 1 km². Parametric OLS regression was then applied to quantify the relative contributions of demographic, while accounting for topographic, accessibility, and climatic factors, to observed forest-cover changes (Table S2). The OLS approach was selected because preliminary analyses indicated that the relationships between these variables and forest-cover change could be appropriately represented using a linear model. The model is formulated as follows:

Δ*FOR_i_* = α_0_ + α_1_.Δ*POP_i_* + α_2_.*BFOR_i_* + α_3_.*BPOP_i_* + α_4_.*SLOPE_i_* + α_5_.*ROAD_i_*

+ α_6_.Δ*TEMP_i_* + α_7_.Δ*RAIN_i_* Eq. S1

where Δ*FORᵢ* denotes the percentage change in forest cover within grid cell *i* between 2017 and 2024, and Δ*POPᵢ* represents the corresponding change in population density (persons/km²). Baseline conditions in 2017 are represented by *BFORᵢ* (forest cover) and *BPOPᵢ* (population density). Topographic characteristics (slope (*SLOPEᵢ*), accessibility (distance to nearest primary road (*ROADᵢ*)), and climatic variables (changes in annual temperature (Δ*TEMPᵢ*) and annual rainfall (Δ*RAINᵢ*))) are included as covariates. All predictor variables demonstrate pairwise correlations below 0.41 (Figure S1), indicating that multicollinearity is not a concern and supporting their concurrent inclusion in the model. The model parameters consist of the intercept coefficient α_0_​ and the explanatory variable coefficients α_1_​, …, α_7_, all of which are estimated during the parameterization process. A negative α₁ coefficient implies that population growth is associated with forest loss, whereas a positive α₁ indicates the population growth is associated with forest regrowth.

B.2. Analysis 2: Migration and changes in forest dependence

Using data from the household survey, we examined how household migration rates shape changes in perceived forest dependency, while accounting for potential confounding factors such as land and livestock ownership and proximity to forest areas. Previous studies conducted in Nepal and other regions have demonstrated that community dependence on forests varies substantially with livelihood characteristics, particularly landholding size and livestock ownership, as forests often serve as critical resources supporting these subsistence activities (Bista 2021). Similarly, the distance from households to forested areas has been shown to influence the degree of forest dependency, as greater distance can limit access to forest resources and thus modify household reliance patterns (Mendako et al. 2022).

To investigate these relationships, we employed a semi-parametric modelling framework based on GAM (Table S2). In our model, the dependent variable (*FDEP_i_*) represents whether a household’s forest dependency has decreased (coded as 1) or has increased or remained unchanged (coded as 0) in the past ten years The principal explanatory variable is the proportion of migrants within household *j* (*MGRT_j_*), calculated as the ratio of household members currently migrated to the total pre-migration household size. In addition, we incorporated a set of control variables to capture household-level characteristics that could potentially confound the relationship between migration and forest dependency. These variables include the total land area owned by the household (*LANDH_j_*), the number of livestock units (*LVSIZE_j_*), the household’s distance to the nearest CF (*CFDIST_j_*), and whether the household holds membership in a CFUG (*CFUG_j_*). Because the first three variables exhibited pronounced right-skewed distributions, we applied natural logarithmic transformations to better approximate normality, reduce the influence of extreme values, and improve the overall model fit.

The data was fitted using the gam package in R (Hastie 2022), applying a logit link to appropriately model the binary response variable. To address potential nonlinear relationships, smooth terms were estimated for predictors exhibiting nonlinearity, enabling the model to capture complex, data-driven associations between explanatory variables and the perceived change in forest dependency. Formally, the model for household *j* can be expressed as:

logit [P(*FDEP_j_* =1)] = β_0_ + β_1_.*MGRT_j_* + β_2_.*CFUG_j_* + *f*_1_(*CFDIST_j_*)+ *f*_2_(*LANDH_j_*)

+ *f*_3_(*LVSIZE_j_*) Eq. S2

The model parameters include the intercept coefficient β_0_, the parametric coefficients β_1_ and β_2_ corresponding to the explanatory variables *MGRT_j_* and *CFUG_j_*, and the functions *f*_1_, *f*_2_, and *f*_3_ which represent the smoothing terms of the continuous covariates *CFDIST_j_*, *LANDH_j_*, and *LVSIZE_j_* respectively. All predictor variables exhibit pairwise correlations below 0.3, supporting their simultaneous inclusion in the model. The parameters are jointly estimated during model fitting. A positive β_1_ suggests that higher levels of household migration increase the likelihood that a household perceives its forest dependency to have declined over recent decade, whereas a negative β_1_ indicates otherwise.

B.3. Analysis 3: Migration, changes in forest dependence, and community participation

Building on the analytical framework that established the relationship between household migration and shifts in forest dependency, this section extends the inquiry to examine how migration and changes in forest dependency subsequently influence levels of community participation in forest-related domains. Because migration decisions are often driven primarily by limited local employment opportunities or the pursuit of better jobs and education elsewhere (Elder et al. 2015; Jones et al. 2018), we include this factor to assess how participation varies across these motivations. To ensure robust inference, we controlled for key household- and context-level factors, including membership in CFUGs, landholding size, livestock holdings, and proximity to the CF area. Community participation was operationalized through three interrelated indicators as mentioned above.

The size of landownership and livestock holdings are widely recognized as important factors of forest participation. Landownership serves as a proxy for livelihood options and opportunity costs, with empirical evidence offering mixed insights on whether larger landholdings promote or constrain participation. Households with greater livestock endowments are often more actively engaged, reflecting enhanced labour capacity and increased demand for forest fodder (Chhetri et al. 2013; Oli & Treue 2015). Proximity to the forest area captures transaction costs and is consistently associated with participation, as households located further away are less likely to attend meetings or engage in collective activities, a pattern observed in both Nepal and Ethiopia (Chhetri et al. 2013; Tadesse et al. 2017). Beyond household and spatial factors, institutional embeddedness, measured through membership in CFUGs, remains a critical determinant of engagement. Membership confers greater access to information, decision-making authority, and associated benefits, which has been consistently linked to higher levels of participation (Oldekop et al. 2018; Bhawana & Race 2020; Smith et al. 2024)

To assess these relationships, we analysed the household survey data using a semi-parametric POLR model with a spline function (Table S3), implemented via the MASS package in R (Venables & Ripley 2023). This modelling approach was chosen for its flexibility in capturing potentially non-linear associations between the set of community participation indicator *m* ∈ {OP=forest operational activities, DM=decision-making, MT=meeting attendance} in household *j* (*PRTCP_mj_*) and both migration patterns and changes in forest dependency (*FDEP_j_*). Migration patterns were represented by households’ outmigration rates (*MGRT_j_*) and unemployment-related outmigration motives (*UNPLY_j_*). We also controlled for key household-level contextual variables, including landholding size (*LANDH_j_*), livestock units (*LVSIZE_j_*), distance to the CF area (*CFDIST_j_*), and household membership in the CFUGs (*CFUG_j_*). Given that the first three variables exhibited strong right-skewness, natural logarithmic transformations were applied to approximate normality, mitigate the influence of extreme values, and enhance overall model performance. The general specification of the model is given as follows:

logit [P(*PRTCP_mj_* ≤ *k*)] = θ*_k_* ꟷ [ζ_1_*_m_*.*FDEP_j_* + ζ_2_*_m_*.*CFUG_j_* + ζ_3_*_m_*.*MGRT_j_* + ζ_4_*_m_*.*UNPLY_j_*

*+ g*_1_*_m_*(*CFDIST_j_*)+ *g*_2_*_m_*(*LANDH_j_*) + *g*_3_*_m_*(*LVSIZE_j_*)] Eq. S3

In this specification, *PRTCP_mj_* represents the ordered level of participation of household *j* in relation to the community participation indicator m. The parameters θ*_k_* denote the threshold values that differentiate the ordered response categories within the model. The variable *FDEP_j_* is a binary indicator capturing whether the forest dependency of household j has decreased *(*1=decreased; 0=remained the same or increased*)*. Similarly, *CFUG_j_* indicates whether household j holds a membership or service position within the CFUG committee, and *UNPLY_j_* indicates whether the household’s migration was primarily driven by unemployment. Coefficients ζ_1_, ζ_2_, ζ_3_, and ζ_4_ are associated with the model's parametric terms, and the functions *g*_1_, *g*_2_, and *g*_3_ capture the smoothing effects of the continuous covariates.

Supplementary Data

_______________________________________________________________________________

Survey Questionnaires

CFUG Name: Id_No:

1. What is the gender of the household head?
   ☐ Male ☐ Female ☐ Other
2. What is the age of the household head (in years)? __________
3. What is your caste/ethnicity? ________________________
4. How many people currently live in your household? __________
5. What is the highest education level (in completed years) of the household head? __________
6. What is the main occupation of the household head?
   ☐ Agriculture ☐ Service ☐ Business ☐ Foreign Employment ☐ Other: ___________

Has it changed in the past 10 years? _________________

1. How much land do you own? (in ropani) __________ Abandoned land _________
2. How many livestock units does your household currently have? __________
3. How many household members aged 16–65 have migrated in the past 5 years? *(If Q9 =NA; jump to Q14)*
4. Within Nepal (internal migration): __________
5. Outside Nepal (international migration): __________
6. Not Applicable

   What are their migration destinations? Please specify locations: ___________________
7. Reason for migration?

☐ Disasters ☐ Resource competition ☐Unemployment ☐ Others __________

11. What is the gender of the migrants?
 ☐ Male ☐ Female ☐ Both

12. Do you receive remittances from any migrated household member?
 ☐ Yes ☐ No

13. How is remittance mostly used? (Tick all that apply)
 ☐ Basic household needs
 ☐ Land/House construction or purchase
 ☐ Agriculture
 ☐ Loan repayment
 ☐ Education
 ☐ Savings

14. What is your primary source of fuel?
 ☐ Firewood ☐ LPG ☐ Electricity

In the case of firewood, what is its main source?
 ☐ Community Forest ☐ Private Forest ☐ Bari/khet ☐ Market purchase

15. In what other ways does your household interact with the forest? (Tick all that apply)
 ☐ Collection of timber for household use
 ☐ Use of forest water sources (springs, streams)
 ☐ Wild fruits and vegetables
 ☐ Collection of NTFPs
 ☐ Ecotourism or income-generating activities
 ☐ Other: _______________________________

16. How far is the community forest from your home? __________mins

17. How often do you visit community forest in a month? __________

18. Has your household's forest dependency changed in the past 10 years?
 ☐ Increased ☐ Decreased ☐ Stayed the same

19. To what extent do the following challenges currently affect your household or community's

ability to manage forests and land?

(Please rate from 1=weak to 5=Very severely)

| Challenge | 1 | | 2 | | 3 | 4 | 5 |
| --- | --- | --- | --- | --- | --- | --- | --- |
| a) Shortage of working-age family members | | ☐ | | ☐ | ☐ | ☐ | ☐ |
| b) Abandonment of farmland or terraces | | ☐ | | ☐ | ☐ | ☐ | ☐ |
| c) Increased human wildlife conflict | | ☐ | | ☐ | ☐ | ☐ | ☐ |
| d) Conflicts over natural resource access | | ☐ | | ☐ | ☐ | ☐ | ☐ |
| f) Other (specify): ____________________ | | ☐ | | ☐ | ☐ | ☐ | ☐ |
|  |  |  |  |  |  |  |  |

20. Has your household experienced Human-Wildlife Conflict (HWC)?

☐ Yes   ☐ No

*If yes:*

*a.* In which year did your household first experience Human-Wildlife Conflict? (separate for

different animals) ______________________________

b. What types of conflicts have you experienced? *(Tick all that apply)*
 ☐ Crop damage ☐ Livestock loss ☐ Property damage ☐ Human injury or threat

c. Which animals were involved in the conflict? *(Tick all that apply)*
 ☐ Monkey ☐ Wild Boar ☐ Porcupine ☐ Bear ☐ Leopard ☐ Others: ____________

d. How much of your land (in ropani or %) has been affected by crop or property damage?
 __________ Ropani or __________ %

e. How frequently do HWC incidents occur in a year?
 ☐ Once or twice ☐ 3–5 times  ☐ More than 5 times  ☐ Seasonal/Annual

21. Are you or a member of your household a member of the Community Forest User Group (CFUG)? ☐ Yes ☐ No

22. How many years has your household been a member of the CFUG? __________

23. How often do you or your family members participate in the following forest activities?

(Please tick one box per row)

| Activity | Never (0) | Sometimes (1) | Frequently (2) |
| --- | --- | --- | --- |
| Forest management activities (cleaning, weeding, harvesting, etc.) | ☐ | ☐ | ☐ |
| Decision-making or planning meetings | ☐ | ☐ | ☐ |
| Executive meetings/general assemblies | ☐ | ☐ | ☐ |

24. Do you feel that your household’s participation in CFUG activities has changed due to

migration?
 ☐ Yes ☐ No ☐ Don’t know

If yes, how has it changed? ______________________________________

25. In the above context, has women’s participation in CFUG changed because of male migration?
 ☐ Increased
 ☐ Stayed the same
 ☐ Decreased

26. In what specific ways has women’s participation in CFUG activities increased due to male

migration? Please describe.

____________________________________________________________

27. Has any household member ever held a leadership position in the CFUG?
 ☐ Yes ☐ No

If yes, which position? ____________________________________

28. Are you (or any family member) willing to participate in CFUG activities in the future?
 ☐ Yes ☐ No ☐ Not sure

If no or not sure, why?
 ☐ Lack of time
 ☐ Lack of active family member(migrant)
 ☐ Gender roles/responsibilities
 ☐ Long distance to CFUG
 ☐ Other: ________________________

29. Changes that you have observed in the community forests due to above reasons:

a) Increased forest fires

b) Invasive plant species spreading

c) More pest or forest disease

d) Lower forest regeneration

e) Others________________

30. How would you rate your level of inclusion in the Community Forest User Group (CFUG)

process in the following areas?

(Please tick one option for each statement)

| Area | 1 | 2 | 3 | 4 | 5 |
| --- | --- | --- | --- | --- | --- |
| a) Participation in meetings and activities | ☐ | ☐ | ☐ | ☐ | ☐ |
| b) Ability to voice concerns or opinions | ☐ | ☐ | ☐ | ☐ | ☐ |
| c) Involvement in actual decision-making processes | ☐ | ☐ | ☐ | ☐ | ☐ |

_______________________________________________________________________________

Supplementary Tables

**Table S1.** Summary of the Community Forest User Groups (CFUGs) surveyed within Sano Sirubari and Thulo Sirubari Wards of Chautara Sangachowkgadhi Municipality, Sindhupalchowk District, Nepal. Data were obtained from the municipal office.

| CFUG Name | Handover date  to community  in BS Nepali calendar  (and *AD*) | CF area  (ha) | Number of households | Total population | Key  Committee  members |
| --- | --- | --- | --- | --- | --- |
| Nepane | 28/05/2050  (*12/09/1993*) | 87 | 124 | 696 | 11 members  (5 women and 6 men) |
| Jalpa | 03/08/2050  (*03/06/1993*) | 38 | 121 | 669 | 9 members  (5 women and 4 men) |
| Rolpakha | 09/03/2052  (*23/06/1995*) | 142 | 277 | 1,930 | 11 members  (5 women and 6 men) |
| Tamakhani | 12/05/2060  (*29/08/2003*) | 105 | 185 | 1,087 | 10 members  (6 women and 4 men) |
| Mahila Deurali | 25/03/2067  (*19/06/2010*) | 5 | 67 | 360 | 11 members  (all women) |

**Table S2.** Variables used in Analysis 1 (population shifts and forest cover change, estimated using OLS) and Analysis 2 (migration and changes in forest dependence, estimated using GAM), including each variable’s notation, definition, measurement, role in the model, expected directional effect on the dependent variable, and corresponding references.

| **Variable** | **Notation**  **(Eq. S1 & Eq. S2)** | **Definition and measurement** | **Variable role in the model** | **Expected effect**  **on the dependent variable** | |
| --- | --- | --- | --- | --- | --- |
|  |  |  |  | **Direction** | **Supporting literature** |
| **Analysis 1: Population shifts and forest cover change (using OLS)** | | | | | |
| Forest cover change | Δ*FOR* | Percentage change in forest cover in each 1 km² grid cell between 2017 and 2024, derived from Dynamic World tree-cover composites | Dependent |  |  |
| Population change | Δ*POP* | Change in population density (persons/km²) between 2017 and 2024, based on WorldPop data rescaled to match official municipal totals | Key explanatory | − | Oldekop et al. (2018); Mather & Needle (2000) |
| Baseline forest cover | *BFOR* | Forest cover in 2017 in each 1 km² grid cell, derived from Dynamic World tree-cover composites | Control | + | ___ |
| Baseline population | *BPOP* | Population density in 2017 in each 1 km² grid cell, based on WorldPop data rescaled to match official municipal totals | Control | − | Lambin & Meyfroidt (2011) |
| Slope | *SLOPE* | Terrain slope in degrees, derived from SRTM elevation data (30 m resolution) | Control | + | Niraula et al. (2013) |
| Distance to primary roads | *ROAD* | Euclidean distance from each grid cell to the nearest primary road, derived from OpenStreetMap data | Control | + | Laurance et al. (2014) |
| Temperature change | Δ*TEMP* | Change in mean annual temperature between 2017 and 2024, derived from ERA5 reanalysis data | Control | − | Panthi et al. (2017) |
| Rainfall change | Δ*RAIN* | Change in total annual precipitation between 2017 and 2024, derived from CHIRPS dataset | Control | + | Panthi et al. (2020) |
| **Analysis 2: Migration and changes in forest dependence (using GAM)** | | | | | |
| Perceived decline in forest dependency | *FDEP* | Binary variable coded as 1 if the household reported a decrease in forest dependency over the past ten years, and 0 otherwise | Dependent |  |  |
| Household migration rate | *MGRT* | Proportion of household members currently migrated relative to total pre-migration household size | Key explanatory | + | Oldekop et al. (2018); Bhawana & Race (2020); Chhetri et al. (2021) |
| CFUG membership | *CFUG* | Binary indicator denoting whether the household is a member of, or holds a service role within, a Community Forest User Group | Control | − | Adhikari et al. (2014); Oli & Treue (2015) |
| Distance to community forest | *CFDIST* | Distance from the household to the nearest community forest area | Control | + | Chhetri et al. (2013); Tadesse et al. (2017) |
| Landholding size | *LANDH* | Total land owned by the household (in Ropani), log-transformed in empirical models to reduce skewness | Control | + | Chhetri et al. (2013); Oli & Treue (2015) |
| Livestock holdings | *LVSIZE* | Total livestock units owned by the household, log-transformed in empirical models | Control | - | Marquardt et al. (2016); Chhetri et al. (2021) |

**Table S3.** Variables used in Analysis 3 (migration, changes in forest dependence, and community participation, estimated using POLR), including each variable’s notation, definition, measurement, role in the model, expected directional effect on the dependent variable, and corresponding references. Three dependent variables capture distinct dimensions of community participation in forest governance: (i) forest operational activities (OP), (ii) decision-making (DM), and (iii) meeting attendance (MT).

| **Variable and role** | **Notation**  **(Eq. S3)** | **Expected direction of effects on the dependent variable** | | | **Rationale for the expected effect on the dependent variable** | **Key supporting literature** |
| --- | --- | --- | --- | --- | --- | --- |
|  |  | **OP** | **DM** | **MA** |  |  |
| ***Key explanatory variable*** | |  |  |  |  |  |
| Household migration rate | *MGRT* | **−** | **−** | **−** | Migration depletes household labour, disrupts intergenerational transmission of forest knowledge, and weakens reciprocity networks underpinning collective action. | Robson & Berkes (2011); Shahi et al. (2022); Bista et al. (2023); Laudari et al. (2024); Benedum et al. (2025) |
| Unemployment-driven migration | *UNPLY* | − | − |  | Households displaced due to the collapse of local livelihoods often experience weakened economic standing and reduced social capital, limiting their capacity for meaningful engagement. However, participation is theorised to be independent of migration motives, with the existing literature does not normally isolate unemployment-driven migration. | Adhikari et al. (2014); Shahi et al. (2022); Cook et al. (2025) |
| Perceived decline in forest dependency | *FDEP* | − | +/− |  | Declining material returns reduce incentive for labour-intensive management. | Adhikari et al. (2014); Chhetri et al. (2021); Shahi et al. (2022); Poudyal et al. (2023); Cook et al. (2025); |
| ***Control variable*** |  |  |  |  |  |  |
| CFUG membership | *CFUG* | + | + | + | Formal membership confers institutional access, information flows, voting rights, and is the mechanical precondition for sanction-enforced attendance. | Adhikari et al. (2014); Oldekop et al. (2018); Bhawana & Race (2020); Smith et al. (2024) |
| Distance to community forest | *CFDIST* | **−** | **−** | **−** | Greater distance raises the marginal cost of engagement across all three dimensions, although sanction-enforcement may partially offset the effect on attendance. | Chhetri et al. (2013); Tadesse et al. (2017); Mendako et al. (2022) |
| Landholding size | *LANDH* | +/− | +/− | +/− | Larger holdings may reflect higher opportunity costs or elite stakes in governance that increased participation. Evidence is mixed across all three dimensions. | Chhetri et al. (2013); Adhikari et al. (2014); Oli & Treue (2015); Baral et al. (2019) |
| Livestock holdings | *LVSIZE* | + | + | +/− | Fodder demand creates a sustained, direct incentive for forest operational activities and a governance stake in rules on forest use. For meeting attendance, effects are weak, since procedural compliance is decoupled from resource-based incentive. | Chhetri et al. (2013); Oli & Treue (2015); Shahi et al. (2022) |

**Table S4**. Results of the Ordinary Least Squares (OLS) regression analyzing the relationship between population changes and forest cover dynamics from 2017 to 2024, controlling topography (slope), accessibility (proximity to roads), climatic change variables (temperature and precipitation), and baseline forest cover and population in 2017, based on spatial datasets at the municipality level (see Eq. S1).

| Variable  (notation in Eq. S1) | Parameter  Estimated coefficient  (95% Confidence Interval (CI)) | | | p-value ǂ | |
| --- | --- | --- | --- | --- | --- |
| Population change (Δ*POP*) | α_1_ | -0.0164 | (-0.0298, -0.0029) | | 0.0175 (●) |
| Baseline forest cover (*BFOR*) | α_2_ | 0.0336 | (0.0013, 0.0659) | | 0.0413 (●) |
| Baseline population (*BPOP*) | α_3_ | 0.0029 | (-0.0009, 0.0067) | | 0.1350 (ns) |
| Slope (*SLOPE*) | α_4_ | 0.1646 | (0.0135, 0.3158) | | 0.0329 (●) |
| Proximity to roads (*ROAD*) | α_5_ | -0.0591 | (-0.2759, 0.1578) | | 0.5917 (ns) |
| Temperature change (Δ*TEMP*) | α_6_ | -3.0124 | (-6.5414, 0.5166) | | 0.0939 (+) |
| Precipitation change(Δ*RAIN*) | α_7_ | -0.0067 | (-0.0210, 0.0075) | | 0.3526 (ns) |

ǂ ● p-value<0.05, + p-value<0.1, ns non-significant

**Table S5.** Results of the Generalized Additive Model (GAM) analyzing the effect of migration (*MGRT*) on household dependence on forest resources (*FDEP*). The model accounts for key covariates, including total land area owned (*LANDH*), livestock holdings (*LVSIZE*), distance to the nearest community forest (*CFDIST*), and membership in a community forest user group (*CFUG*) see Eq. S2). Estimated coefficients are reported for parametric terms only, while significance levels (p-values) are provided for both the parametric and smoothing terms. The functional form of the smoothing term is shown in Figure 4.

| Parametric or smoothing term | Variable  (notation in Eq. S2) | Parameter | | | p-value ǂ |
| --- | --- | --- | --- | --- | --- |
|  |  | Estimated coefficient (95% CI) | | |  |
| Parametric | Migration rates (*MGRT*) | β_1_ | 0.050 | (0.004, 0.107) | 0.048 (●) |
|  | CFUG membership (*CFUG*) | β_2_ | 0.769 | (-1.362, 3.265) | 0.497 (ns) |
| Smoothing | **Distance to CF (*CFDIST*)** | *f*_1_ |  |  | 0.032 (●) |
|  | **Land ownership (*LANDH*)** | *f*_2_ |  |  | 0.049 (●) |
|  | **Livestock holdings (*LVSIZE*)** | *f*_3_ |  |  | 0.981 (ns) |

ǂ ● p-value<0.05, ns non-significant

Supplementary Figures


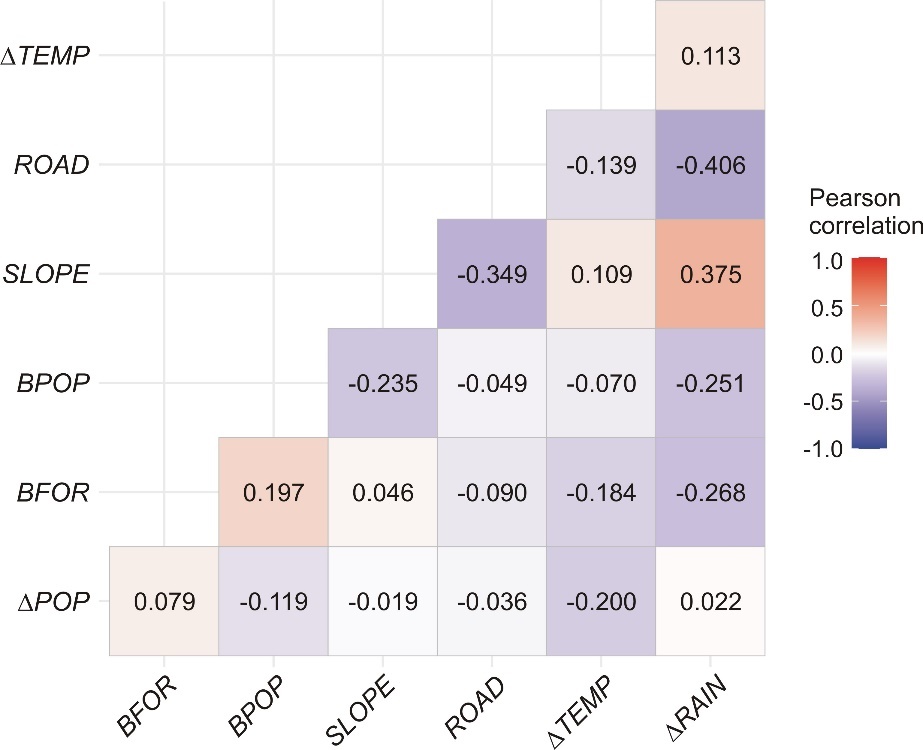


**Figure S1.** Correlogram showing the correlations among predictor variables used to explain changes in forest cover in the Generalized Additive Model (GAM) (Eq. S1). The predictors include changes in population density per km² (Δ*POP*), baseline forest cover (*BFOR*) and population in 2017 (*BPOP*), slope (*SLOPE*), distance to the nearest primary road (*ROAD*), and changes in annual temperature (Δ*TEMP*) and rainfall between 2017 and 2024 (Δ*RAIN*).

Supplementary References

Adhikari S, Kingi T & Ganesh S (2014) Incentives for community participation in the governance and management of common property resources: The case of community forest management in Nepal. For Policy Econ 44: 1–9.

Agarwal, B. (2001) Participatory exclusions, community forestry, and gender: An analysis for South Asia and a conceptual framework. World Dev 29: 1623–1648.

Baral S, Meilby H & Chhetri BBK (2019) The contested role of management plans in improving forest conditions in Nepal’s community forests. Int For Rev 21: 37–50.

Benedum ME, Cook NJ & Vallury S (2025) Remittance income weakens participation in community-based natural resource management. Ecol Soc 30: 34.

Bhawana KC & Race D (2020) Outmigration and land-use change: A case study from the middle hills of Nepal. Land 9: 2.

Bista RB (2021) Livelihood dependency and inequality in community forests of Nepal. Econ J Nepal 44: 37–53.

Bista R, Graybill S, Zhang Q et al. (2023) Influence of rural out-migration on household participation in community forest management? Evidence from the Middle Hills of Nepal. Sustainability 15: 2185.

Brown CF, Brumby SP, Guzder-Williams B et al. (2022) Dynamic World: Near real-time global 10 m land use/land cover mapping. Sci Data 9: 251.

Chhetri BBK, Johnsen FH, Konoshima M & Yoshimoto A (2013) Community forestry in the hills of Nepal: Determinants of user participation in forest management. For Pol Econ 30: 6–13.

Chhetri R, Yokying P, Smith A et al. (2021) Forest, agriculture, and migration: contemplating the future of forestry and agriculture in the middle-hills of Nepal. J Peasant Stud 50: 411–433.

Cook NJ, Khatri DB, Poudel DP et al. (2025) Dropping out of environmental governance: Why Nepal’s community-based forestry program is losing participants. Elementa: Sci Anthropocene 13: 00059.

Dhungana N, Lee CH, Khadka C et al. (2024). Evaluating Community Forest User Groups (CFUGs)’ performance in managing community forests: A case study in Central Nepal. Sustainability 16: 4471.

Dutt S, Batar AK, Sulik S & Kunz M (2024) Forest ecosystem on the edge: Mapping forest fragmentation susceptibility in Tuchola Forest, Poland. Ecol Indic 161: 111980.

Elder S, De Haas H, Principi M & Schewel K (2015) *Youth and Rural Development: Evidence from 25 School-to-Work Transition Surveys.* Geneva: International Labour Organization.

Farr TG, Rosen PA, Caro E et al. (2007) The Shuttle Radar Topography Mission. Rev Geophys 45: RG2004.

Food and Agriculture Organization of the United Nations - FAO (2016) The state of food and agriculture: Climate change, agriculture and food security. FAO.

Funk C, Peterson P, Landsfeld M et al. (2015) The climate hazards infrared precipitation with stations (CHIRPS): A new environmental record for monitoring extremes. Sci Data 2, 150066.

Hastie TJ (2017) *Generalized Additive Models*. Routledge.

Hastie TJ (2022) gam: Generalized Additive Models (R package version 1.20).

Hersbach H, Bell B, Berrisford P et al. (2020) The ERA5 global reanalysis. Q J Roy Meteorol Soc 146: 1999–2049.

Intergovernmental Panel on Climate Change - IPCC (2023) Climate Change 2023: Synthesis Report. Contribution of Working Groups I, II and III to the Sixth Assessment Report of the Intergovernmental Panel on Climate Change (H. Lee & J. Romero, Eds.). IPCC.

Jones JPG, Mandimbiniaina R, Kelly R et al. (2018) Human migration to the forest frontier: Implications for land use change and conservation management. Geo: Geogr Environ 5: e00050.

Lambin EF & Meyfroidt P (2011) Global land use change, economic globalization, and the looming land scarcity. Proc Natl Acad Sci USA 108: 3465–3472.

Láng-Ritter J, Keskinen M & Tenkanen H (2025) Global gridded population datasets systematically underrepresent rural population. Nat Comm 16: 2170.

Laudari HK, Sapkota LM, Maraseni T et al. (2024) Community forestry in a changing context: A perspective from Nepal’s mid-hill. Land Use Policy 138: 107018.

Laurance W, Clements G, Sloan S. et al. (2014) A global strategy for road building. Nature 513: 229–232.

Leyk S, Gaughan AE, Adamo SB et al. (2019) The spatial allocation of population: a review of large-scale gridded population data products and their fitness for use. Earth Syst Sci Data 11: 1385–1409.

Marquardt K, Khatri D & Pain A (2016) REDD+, forest transition, agrarian change and ecosystem services in the hills of Nepal. Hum Ecol 44: 229–244.

Mather AS & Needle CL (2000) The relationships of population and forest trends. Geogr J 166: 2–13.

Mendako RK, Tian G & Matata PM (2022) Identifying socioeconomic determinants of households’ forest dependence in the Rubi-Tele Hunting Domain, DR Congo: A logistic regression analysis. Forests 13: 1706.

Niraula RR, Gilani H, Pokharel BK & Qamer FM (2013) Measuring impacts of community forestry program through repeat photography and satellite remote sensing in the Dolakha district of Nepal. J Environ Manage 126: 20–29.

Oldekop JA, Sims KRE, Whittingham MJ & Agrawal A (2018) An upside to globalization: International outmigration drives reforestation in Nepal. Glob Environ Change 52: 66–74.

Oli BN & Treue T (2015) Determinants of participation in Community Forestry in Nepal. Int For Rev 17: 311–321.

Panthi S, Bräuning A, Zhou ZK & Fan ZX (2017) Tree rings reveal recent intensified spring drought in the central Himalaya, Nepal. Glob Planet Change 157: 50–59.

Panthi S, Fan ZX, van der Sleen P & Zuidema PA (2020) Long-term physiological and growth responses of Himalayan fir to environmental change are mediated by mean climate. Glob Chang Biol 26: 1649–1664.

Pariyar SB, Bhattarai S & Dhakal S (2025) Defining indicators of active community forest management: basis for assessing sustainable forest management in Nepal. Discover For 1: 22.

Poudyal BH, Khatri DB, Paudel D et al. (2023) Examining forest transition and collective action in Nepal’s community forestry. Land Use Policy 134: 106872.

Robson J & Berkes F (2011) How does out-migration affect community institutions? A study of two indigenous municipalities in Oaxaca, Mexico. Hum Ecol 39: 179–190.

Rudel TK, Coomes OT, Moran E et al. (2005) Forest transitions: Towards a global understanding of land use change. Glob Environ Change 15: 23–31.

Shahi N, Bhusal P, Paudel G & Kimengsi JN (2022) Forest–people nexus in changing livelihood contexts: Evidence from community forests in Nepal. Trees For People 8: 100223.

Smith AC, Hajjar R, Kanel KR et al. (2024) Out-migration, agricultural abandonment, and community forest management: Drivers of afforestation in privately managed land in Nepal. Appl Geogr 167: 103275.

Tadesse S, Woldetsadik M & Senbeta F (2017) Forest users’ level of participation in a participatory forest management program in southwestern Ethiopia. Forest Sci Technol 13: 164–173.

Tatem AJ (2017) WorldPop, open data for spatial demography. Sci Data 4: 170004.

Venables WN & Ripley BD (2023) MASS: Support functions and datasets for Venables and Ripley’s MASS (R package version 7.3-65).

Venter ZS, Barton DN, Chakraborty T et al. (2022) Global 10 m land use land cover datasets: A comparison of Dynamic World, World Cover and Esri Land Cover. Remote Sens 14: 4101.

Zhao W, Zhong X, Li X et al. (2025) Automatic mapping of 10 m tropical evergreen forest cover in Central African Republic with Sentinel-2 Dynamic World dataset. Remote Sens 17: 722.
